# A Randomized Trial of Drug Route in Out-of-Hospital Cardiac Arrest

**DOI:** 10.1056/NEJMoa2407780

**Published:** 2024-10-31

**Authors:** Keith Couper, Chen Ji, Charles D Deakin, Rachael T Fothergill, Jerry P Nolan, John B Long, James M Mason, Felix Michelet, Chloe Norman, Henry Nwankwo, Tom Quinn, Anne-Marie Slowther, Michael A Smyth, Kath R Starr, Alison Walker, Sara Wood, Steve Bell, Gemma Bradley, Martina Brown, Shona Brown, Emma Burrow, Karl Charlton, Andrew Claxton, Victoria Dra’gon, Christine Evans, Jakob Falloon, Theresa Foster, Justin Kearney, Nigel Lang, Matthew Limmer, Adam Mellett-Smith, Joshua Miller, Carla Mills, Ria Osborne, Nigel Rees, Robert E.S. Spaight, Gemma L Squires, Belinda Tibbetts, Michelle Waddington, Gregory A. Whitley, Jason V. Wiles, Julia Williams, Sarah Wiltshire, Adam Wright, Ranjit Lall, Gavin D Perkins, on behalf of the PARAMEDIC-3 collaborators

**Affiliations:** Warwick Medical School, Clinical Trials Unit, University of Warwick, Coventry, UK; North Eest Ambulance Service NHS Trust, Newcastle Upon Tyne, UK; Critical Care Unit, University Hospitals Birmingham NHS Foundation Trust, Birmingham, UK; Warwick Medical School, Clinical Trials Unit, University of Warwick, Coventry, UK; South Central Ambulance Service NHS Foundation Trust, Bicester, UK; University Hospital Southampton NHS Foundation Trust, Southampton, UK; London Ambulance Service NHS Trust, London, UK; Warwick Medical School, Clinical Trials Unit, University of Warwick, Coventry, UK; Department of Anaesthesia, Royal United Hospitals Bath NHS Foundation Trust, Bath, UK; University of Bristol, Bristol, UK; Warwick Medical School, Clinical Trials Unit, University of Warwick, Coventry, UK; Warwick Medical School, Clinical Trials Unit, University of Warwick, Coventry, UK; Warwick Medical School, Clinical Trials Unit, University of Warwick, Coventry, UK; Warwick Medical School, Clinical Trials Unit, University of Warwick, Coventry, UK; Warwick Medical School, Clinical Trials Unit, University of Warwick, Coventry, UK; Kingston University, London, UK; Warwick Medical School, Clinical Trials Unit, University of Warwick, Coventry, UK; Warwick Medical School, Clinical Trials Unit, University of Warwick, Coventry, UK; Critical Care Unit, University Hospital Coventry and Warwickshire NHS Trust, Coventry, UK; Warwick Medical School, Clinical Trials Unit, University of Warwick, Coventry, UK; North West Ambulance Service NHS Trust, Bolton, UK; West Midlands Ambulance Service University NHS Foundation Trust, Brierley Hill, UK; Warwick Medical School, Clinical Trials Unit, University of Warwick, Coventry, UK; East of England Ambulance Service NHS Trust, Cambridge, UK; South East Coast Ambulance Service NHS Foundation Trust, Crawley, UK; South Central Ambulance Service NHS Foundation Trust, Bicester, UK; North West Ambulance Service NHS Trust, Bolton, UK; North Eest Ambulance Service NHS Trust, Newcastle Upon Tyne, UK; Critical Care Unit, University Hospitals Birmingham NHS Foundation Trust, Birmingham, UK; South Central Ambulance Service NHS Foundation Trust, Bicester, UK; South Central Ambulance Service NHS Foundation Trust, Bicester, UK; West Midlands Ambulance Service University NHS Foundation Trust, Brierley Hill, UK; London Ambulance Service NHS Trust, London, UK; East of England Ambulance Service NHS Trust, Cambridge, UK; London Ambulance Service NHS Trust, London, UK; Devon Air Ambulance, Exeter, UK; North Eest Ambulance Service NHS Trust, Newcastle Upon Tyne, UK; London Ambulance Service NHS Trust, London, UK; West Midlands Ambulance Service University NHS Foundation Trust, Brierley Hill, UK; Welsh Ambulance Services University NHS Trust, Cwmbran, Wales, UK; South Western Ambulance Service NHS Foundation Trust, Exeter, UK; Welsh Ambulance Services University NHS Trust, Cwmbran, Wales, UK; East Midlands Ambulance Service NHS Trust, Nottingham, UK; East Midlands Ambulance Service NHS Trust, Nottingham, UK; Devon Air Ambulance, Exeter, UK; North West Ambulance Service NHS Trust, Bolton, UK; East Midlands Ambulance Service NHS Trust, Nottingham, UK; West Midlands Ambulance Service University NHS Foundation Trust, Brierley Hill, UK; South East Coast Ambulance Service NHS Foundation Trust, Crawley, UK; South Western Ambulance Service NHS Foundation Trust, Exeter, UK; Emergency Department, Harrogate and District NHS Foundation Trust, Harrogate, UK; Warwick Medical School, Clinical Trials Unit, University of Warwick, Coventry, UK; Warwick Medical School, Clinical Trials Unit, University of Warwick, Coventry, UK; Critical Care Unit, University Hospitals Birmingham NHS Foundation Trust, Birmingham, UK

## Abstract

**Background:**

In out-of-hospital cardiac arrest, the effectiveness of drugs, such as epinephrine, is highly time-dependent. The intraosseous, as compared with intravenous, drug route may facilitate more rapid drug administration, but its effect on clinical outcomes is uncertain.

**Methods:**

In a multicenter, open-label randomized trial across 11 emergency medical systems in the United Kingdom, paramedics randomly assigned adults in cardiac arrest requiring drug therapy to an intraosseous-first or intravenous-first vascular access strategy. The primary outcome was survival at 30-days, with key secondary outcomes including favorable neurological outcome at hospital discharge (modified Rankin Scale score ≤3, range 0-6) and return of spontaneous circulation. We made no adjustment for multiplicity.

**Results:**

Among 6082 randomized participants, 30-day survival occurred in 137 of 3030 (4.5%) in the intraosseous group and 155 of 3034 (5.1%) in the intravenous group (adjusted odds ratio 0.945, 95% confidence interval 0.676-1.322, p=0.741). A favorable neurological outcome at hospital discharge occurred in 80/2994 (2.7%) and 85/2986 (2.8%) in the intraosseous and intravenous groups, respectively (adjusted odds ratio, 0.914, 95% CI 0.567-1.474) and return of spontaneous circulation at any time in 1092/3031 (36.0%) and 1186/3035 patients (39.1%) (adjusted odds ratio, 0.863; 95% confidence interval, 0.765 to 0.974). During the trial, one adverse event was reported, which occurred in the intraosseous group.

**Conclusions:**

In adults with out-of-hospital cardiac arrest requiring drug therapy, an intraosseous-first strategy did not improve the rate of 30-day survival. (Funded by the UK National Institute for Health and Care Research; Trial registration: ISRCTN14223494).

## Introduction

Recent trials have explored the clinical effectiveness of cardiac arrest drugs.^1,2^ The effects of which are highly time-dependent, suggesting earlier drug administration may improve outcomes.^3,4^ Securing intravenous access in out-of-hospital cardiac arrest is challenging, because of environmental and patient factors. In previous trials, time from the emergency call to drug administration has ranged from 16 to 21-minutes.^1,2,5^

In observational studies and one small randomized trial, the intraosseous, compared with the intravenous route, facilitated more rapid drug administration, particularly when the proximal tibial site is used.^6,7^ Observational studies comparing intraosseous and intravenous drug administration in cardiac arrest report similar or worse outcomes in patients receiving intraosseous drugs, but these studies are challenging to interpret as intraosseous access is typically attempted after a failed intravenous access attempt confounding findings because of resuscitation time bias.^8–10^ International resuscitation guidelines recommend peripheral intravenous access as the primary vascular access route,^11,12^ but studies show increasing use of intraosseous access (up to 60%) in some systems.^5,13–15^

Given ongoing uncertainty as to the optimal drug route in adult cardiac arrest, the International Liaison Committee on Resuscitation highlighted the urgent need for randomized trials to evaluate the clinical effectiveness of the intraosseous access route.^16^ In response, we conducted the PARAMEDIC-3 trial to determine the clinical effectiveness of an intraosseous-first compared with an intravenous-first strategy for adult out-of-hospital cardiac arrest.

## Methods

### Trial design and oversight

PARAMEDIC-3 was a pragmatic parallel group, open-label, randomized trial conducted across 11 UK emergency medical systems (10 NHS ambulance services and one standalone air ambulance service) between November 2021 and July 2024. The trial was prospectively registered (ISRCTN14223494). The protocol, developed by the trial investigators, has been published previously and is available, together with the statistical analysis plan, in the Supplementary Materials.^17^ The trial protocol was approved by the South Central–Oxford C Research Ethics Committee (21/SC/0178) and the Health Research Authority Confidentiality Advisory Group (20/CAG/0092). Due to the time-critical nature of treatment in cardiac arrest, the Research Ethics Committee approved a process of initial enrolment without consent in accordance with local legislation (details provided in the supplementary appendix). Consent for ongoing data collection was subsequently sought from survivors or a proxy decision-maker if the individual lacked capacity.

The trial was funded by the National Institute for Health and Care Research Health Technology Assessment programme, who had no role in trial design, data collection, data analysis, or the preparation of the manuscript. The trial was sponsored by the University of Warwick and co-ordinated by Warwick Clinical Trials Unit. An independent Trial Steering Committee (TSC) and Data Monitoring and Ethics Committee provided oversight. The study adhered to Good Clinical Practice guidelines, local regulations, and the ethical principles described in the Declaration of Helsinki. Trial statisticians (CJ, FM, RL) had full access to trial data and assumed responsibility for the data integrity, completeness and accuracy of the data analysis, and trial fidelity to the protocol. The paper was drafted by the first author; all authors reviewed the manuscript and approved its submission. There were no confidentiality agreements between the sponsor and authors. This paper reports the primary outcome, pre-hospital outcomes, neurological function at hospital discharge, and hospital length of stay. Follow-up at 3-months and 6-months, together with health economic, quality of life, intraosseous access site and critical care length of stay outcomes will be reported separately.

## Patient Population

Adults (≥ 18 years of age), who sustained an out-of-hospital cardiac arrest, were attended by trial-trained paramedics and who required vascular access for drug administration during ongoing cardiopulmonary resuscitation were eligible for the trial. We excluded individuals with known or apparent pregnancy.

## Randomization And Treatment

Participating emergency medical systems delivered resuscitation in accordance with current resuscitation guidelines (see supplementary materials). In the UK, paramedics are trained to provide advanced life support, including manual defibrillation, advanced airway management, drug therapy, and vascular access. Resuscitation may be terminated by paramedics in accordance with recognized criteria. As intravenous and intraosseous access are core skills for UK paramedics, additional training was not required to participate in the trial.

At the time a patient was identified as requiring vascular access, they were randomized in a 1:1 ratio to the intraosseous or intravenous group using a sequentially numbered, tamper-proof, opaque envelope system. This system ensured the randomization process did not delay time-critical interventions. The sequence, created by the trial statisticians, was stratified by site. Envelopes were packed centrally at Warwick Clinical Trials Unit before distribution to sites. To ensure allocation concealment, paramedics opened envelopes only once they had confirmed patient eligibility. At the point that the envelope was opened, the patient was categorized as being randomized.

The trial randomization determined the initial vascular access attempt strategy. If the paramedic could not obtain vascular access by that allocated route within two attempts, the route of subsequent vascular access attempts was determined by the treating paramedic. The anatomical location of both intraosseous and intravenous cannulae was decided by the treating paramedic. Once vascular access was obtained, it was expected that all cardiac arrest drugs were given by that route.

The randomized vascular access route was used until return of spontaneous circulation, termination of resuscitation, an established vascular access was dislodged, or hospital arrival. Treatment following hospital handover was determined by the hospital’s clinical team, informed by international guidelines.^11^ Data were collected by each site in accordance with standardised international definitions.^18^

## Outcomes

The primary trial outcome was survival at 30-days. The secondary outcomes were: (i) any return of spontaneous circulation following randomization, (ii) sustained return of spontaneous circulation on transfer of care to medical staff at the receiving hospital, (iii) survival at hospital discharge, 3-months and 6-months, (iv) time to return of spontaneous circulation, (v) hospital/critical care length of stay, (vi) neurological function (measured by modified Rankin Scale) at hospital discharge, 3-months and 6-months, and (vii) health-related quality of life (measured by the EQ-5D-5L questionnaire) at 3-months and 6-months. The modified Rankin Scale is a seven point scale ranging from 0 (no symptoms) to 6 (death), with a score of ≤3 representing a favorable neurological outcome.^19^ Adverse and serious adverse events were recorded until hospital discharge. The open label nature of the trial precluded blinding of outcome assessors.

## Statistical Analysis

We planned to recruit 15,000 patients. Based on PARAMEDIC2 trial data, 14972 patients were required to detect a difference in the 30-day survival status of 1% (3.2% to 4.2%; 5% significance level; 90% power).^1^ Parameters used in the sample size calculation are described in the protocol. Two formal interim analyses were planned to assess efficacy or harm during the trial (10% and 50% data availability). The O’Brien and Fleming alpha spending method was adopted to develop stopping boundaries and these boundaries preserved the type 1 error rate.^20^

All analyses were carried out using an intention-to-treat approach.^21^ Categorical outcomes, including the primary outcome, were analysed using logistic regression models and presented as odds ratios with 95% confidence intervals. The primary analysis was the adjusted analysis, with adjustments made for age, sex, witness status, bystander CPR, initial rhythm, time from emergency call to drug administration, and etiology. We performed two post-hoc sensitivity analyses to mitigate any causal association between time of drug administration and outcome, namely replacing time to drug administration with response time and removal of time to drug administration. To address the potential overestimation of odds ratios, we report risk differences as a post-hoc analysis. Continuous outcomes were analyzed with linear regression models and time-to-event outcomes were analyzed with cox regression models. There was no indication of violation of the proportional hazard assumption using the Kolmogorov-type supremum test. To prevent multiplicity in hypothesis testing, only the primary outcome was assessed using statistical tests.

For the primary outcome, an Estimand framework^22^ was specified for two intercurrent events as sensitivity analyses: (i) discontinuation of treatment before initiation of the allocated treatment, and (ii) treatment crossover, analyzed using inverse probability censoring weighted methods.^23^ Crossover was defined as the use of the non-randomized drug route prior to two unsuccessful attempts at the randomized route. Missing data were assessed using multiple imputation by chained equations and tipping point analyses. ^24^ The sensitivity of the primary outcome results was tested using the Fragility index.^25^ The Kaplan-Meier curve was plotted for the survival time to 30-days.

Pre-specified sub-group analyses included: age, sex, witness status, bystander CPR, initial rhythm, time from emergency call to ambulance arrival, and etiology. Logistic regression models were fitted for both continuous and categorical sub-group variables. No adjustments were made for multiple hypothesis tests. Data management and analysis was performed with Statistical Analysis Systems (SAS), version 9.4.

## Results

Recruitment was slower than expected and stopped prematurely at the end of the funded recruitment period (1^st^ July 2024) when 6096 participants had been recruited and prior to the second formalised interim analysis. Trial investigators were blinded to study data when the decision to stop recruitment was made. This decision was supported by the TSC and agreed by the study sponsor.

### Patients and interventions

Between November 2021 and July 2024, 10723 patients were screened for eligibility of which 6096 were randomized. Of these, 14 patients were randomized in error. The remaining 6082 patients were assigned to the intraosseous group (n=3040) or intravenous group (n=3042). Patient flow is shown as Figure 1.

Participant baseline characteristics were balanced (Table 1). The representativeness of trial participants is summarized in the supplementary appendix. Key time intervals and interventions are summarized in table 2. From the emergency call, median time to epinephrine administration was 24.0 (IQR 19.0-30.0) minutes. Crossover, defined as the use of the non-randomized drug route prior to two unsuccessful attempts at the randomized route, occurred in 528 (8.7%) participants.

### Primary and secondary outcomes

Primary outcome data were available for 3030 (99.7%) participants in the intraosseous group and 3034 (99.7%) participants in the intravenous group. Survival at 30-days occurred in 137 of 3030 (4.5%) in the intraosseous group and 155 of 3034 (5.1%) in the intravenous group (adjusted odds ratio, 0.945, 95% confidence interval (CI) 0.676-1.322, p=0.741). Results were similar in the unadjusted analysis.

The proportion of patients who survived to hospital discharge with a favorable neurological outcome was 80 of 2994 (2.7%) in the intraosseous group and 85 of 2986 patients (2.8%) in the intravenous group (adjusted odds ratio, 0.914, 95% CI 0.567-1.474). The proportion of patients who achieved return of spontaneous circulation at any time was 1092 of 3031 (36.0%) in the intraosseous group and 1186 of 3035 patients (39.1%) in the intravenous group (adjusted odds ratio, 0.863; 95% CI 0.765-0.974). The proportion who achieved a sustained return of spontaneous circulation and other secondary outcomes are presented in Table 3.

The results for the primary outcome were consistent across prespecified sub-groups (figure 2; figure S5) and sensitivity analyses (tables S10-S13; figure S7).

### Adverse events

One adverse event was reported, whereby a patient in the intraosseous group reported ongoing mild leg pain during certain activities (table three; tables S14 to S16). The event was not deemed to be serious.

## Discussion

In this trial, the use of an intraosseous strategy for vascular access and drug administration in cardiac arrest, compared with an intravenous strategy, did not result in a significantly higher rate of 30-day survival. We observed no apparent difference between groups for favorable neurological outcome at hospital discharge. In the intraosseous strategy group, the rate of return of spontaneous circulation appeared to be lower.

Our trial hypothesis for the superiority of the intraosseous strategy route was that it would facilitate more rapid administration of epinephrine, which would improve 30-day survival through reducing time to return of spontaneous circulation thereby minimizing the hypoxic-ischemic damage that is the main cause of death following cardiac arrest.^26^ This was based on previous studies showing reduced time to drug administration in patients where initial vascular access attempts are made via the intraosseous route, particularly when the proximal tibial route is chosen.^6,7^ In contrast to these previous studies, we found that an intraosseous-first strategy did not reduce time to drug administration.

Despite similarities in time to drug administration, the rate of return of spontaneous circulation in the intraosseous group appeared to be lower, suggesting drug efficacy was influenced by administration route. There are several potential explanations for this. First, intraosseous cannulae might be incorrectly positioned, leading to sub-optimal drug absorption. Whilst we were unable to assess this in our trial, previous studies suggest that intraosseous cannulae are prone to both sub-optimal placement and dislodgement.^6,27^ Second, time to peak drug concentration and the maximal drug concentration may be inferior to the intravenous route, even when the intraosseous cannula is optimally placed. Animal studies suggest proximal humerus intraosseous placement may be better and the proximal tibial placement worse, compared with the peripheral intravenous route, in time to achieve maximal drug concentration.^9^ However, this potential advantage of the proximal humerus, compared with the proximal tibial site, may be off-set by lower success rates, higher dislodgement rates, and longer time to successful placement.^6^ Third, it has been postulated that delivery to the central circulation of lipophilic drugs, such as amiodarone, may be worse when administered by the intraosseous route.^28^

Our results build on the recently published Taiwanese VICTOR trial, a cluster randomized trial where 1771 adult out-of-hospital cardiac arrest patients were randomized to either proximal humeral intraosseous access or upper-limb intravenous access.^29^ Consistent with our trial, the VICTOR trial reported that the intraosseous route did not improve the rate of survival to hospital discharge or reduce time to drug administration.

In our trial, overall median time from emergency call to drug administration was 24 minutes and 30-day survival was 4.8%. Our time to drug administration is comparable to the VICTOR trial and only slightly longer than other randomized trials of cardiac arrest drug interventions, where times have ranged from 16 to 21 minutes.^1,2,5,29^ The overall rate of 30-day survival in the UK is similar to other regions, including areas of North America, Europe, and Asia.^30^ Our target population was patients requiring drug therapy, such that patients in whom initial resuscitation attempts were successful and who have the best outcomes were ineligible for the trial.^1^

Our trial, the largest currently planned, was terminated early because of lower-than-anticipated recruitment, so is under-powered to detect a 1% difference between groups for the primary outcome. Trial investigators, blinded to trial data, made the decision to terminate recruitment, and this coincided with the end of the funded recruitment period. Subsequent analyses show that when this decision was made, effect estimates for the primary outcome were stable, making it unlikely that continuing to the original sample size would have materially influenced trial findings (figure S9).

Our trial has several additional limitations. We did not collect information on resuscitation quality, because of the pragmatic nature of the trial and the challenges of collecting these data. We did not protocolize or collect information on hospital-based post-resuscitation care, although we would expect this care to be consistent between arms in accordance with the UK adoption of international guidance.^31^ The nature of the trial precluded blinding of pre-hospital care providers, but this is unlikely to have introduced performance bias because of the protocolized nature of cardiopulmonary resuscitation, particularly in relation to decisions to terminate resuscitation attempts. Our pre-specified adjusted analyses included time to drug administration as a covariate, which was hypothesised as a potential mediator of effect of the intervention. The absence of a time difference and sensitivity analyses performed suggests this did not materially influence study findings. The main reason why screened patients were not randomized was pre-existing vascular access, which likely occurred most frequently where a non-trial-trained paramedic arrived on scene and secured vascular access, before the arrival of a trial-trained paramedic. It is unlikely this introduced selection bias or influenced the generalizability of our findings as ambulance resources are allocated by a central control room, based on availability and location. In contrast to other trials, we did not protocolize the anatomical location of intraosseous cannulae.^29,32^ This reflects the pragmatic nature of our trial, whereby paramedics select the anatomical location of vascular access based on personal preference and patient characteristics, informed by the available evidence.

In conclusion, for adults with out-of-hospital cardiac arrest requiring drug therapy, an intraosseous-first strategy did not improve the rate of 30-day survival.

Disclosure forms provided by the authors are available with the full text of this article at NEJM.org.

## Supplementary Material

supplement

## Figures and Tables

**Figure one:**
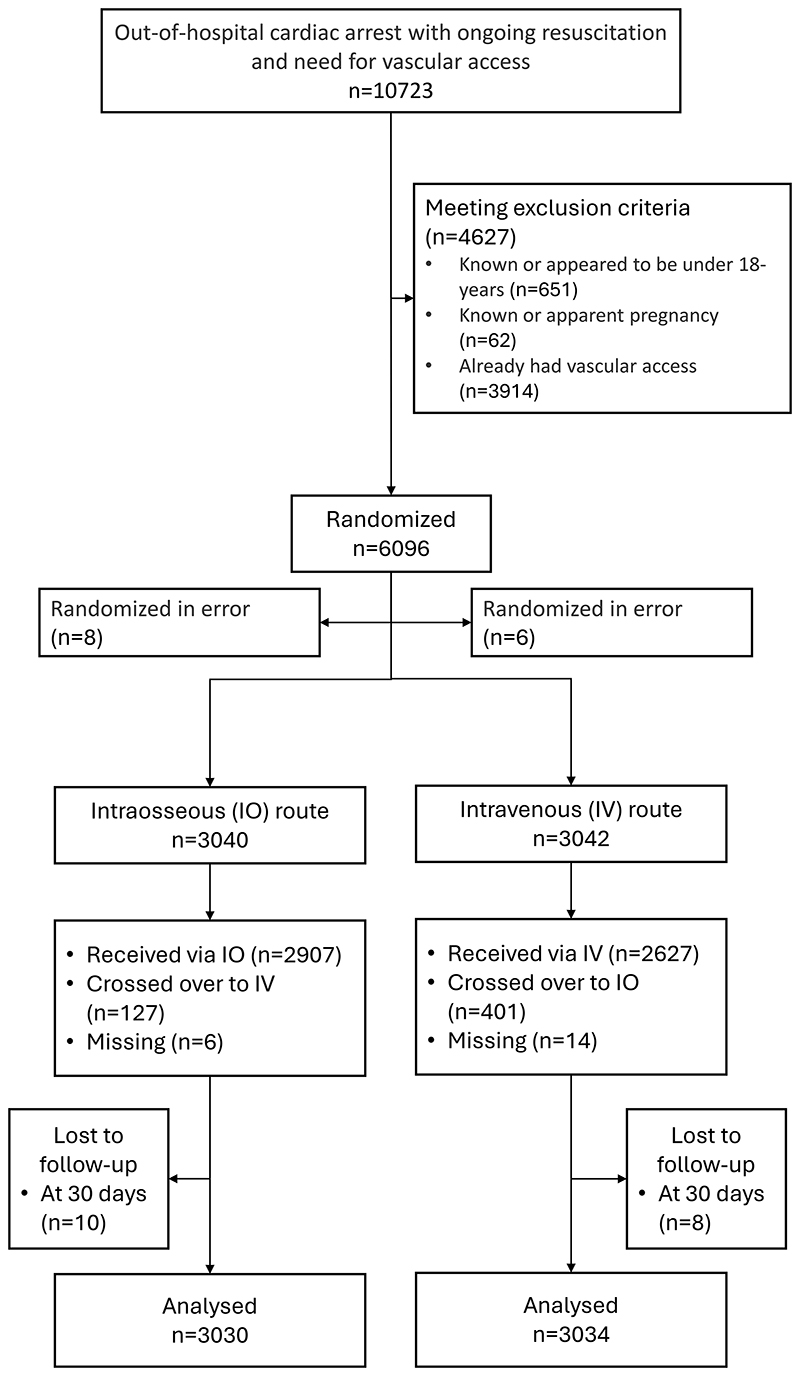
enrolment and outcomes Note: 3914 patients were excluded due to pre-existing vascular access. This likely occurred most frequently where a paramedic not trained in the trial protocol arrived on scene and secured vascular access, before the arrival of a trial-trained paramedic. Crossover is defined as the use of the non-randomized drug route prior to two unsuccessful attempts at the randomized route.

**Figure two:**
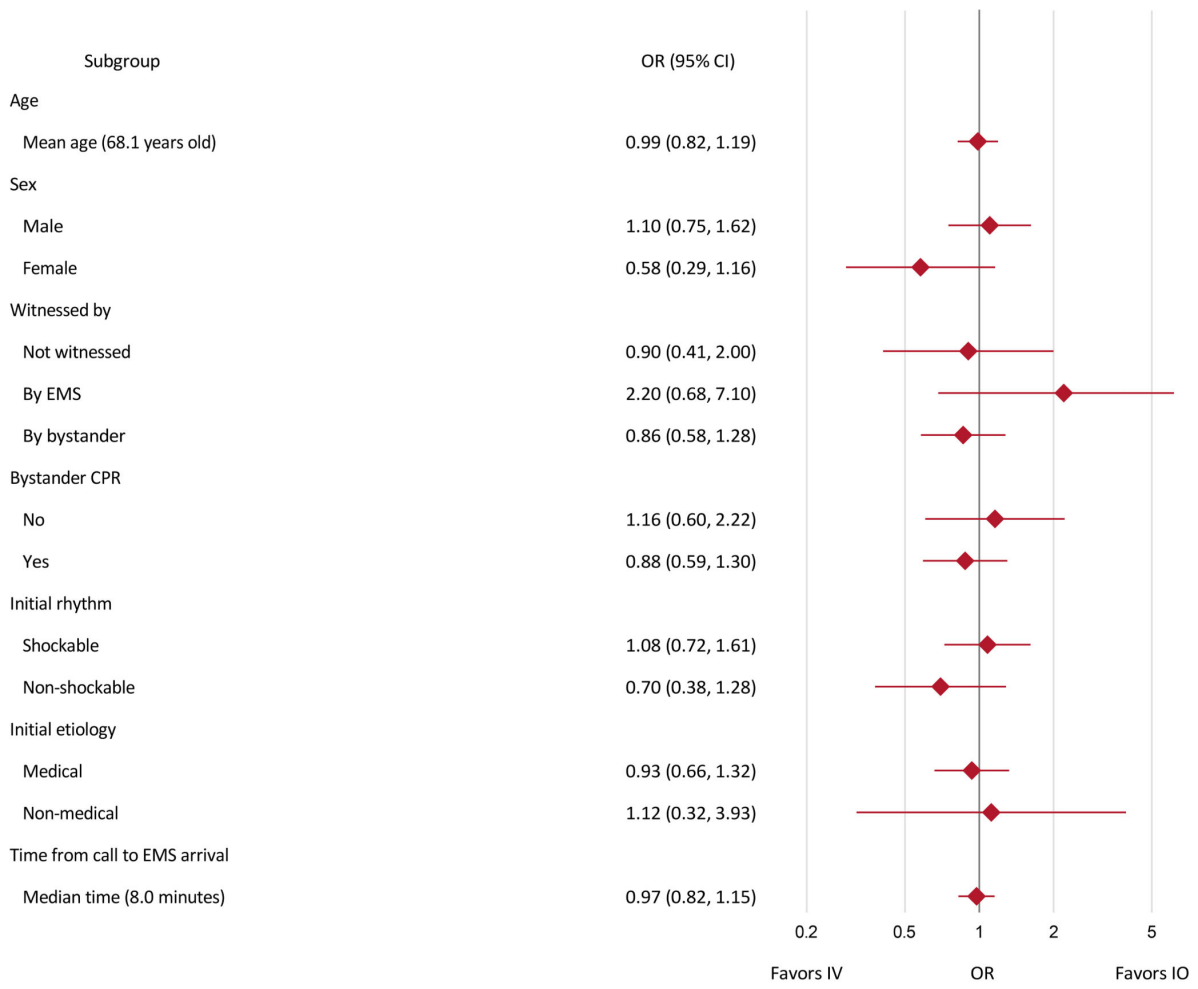
Summary of adjusted subgroup analyses for primary outcome Note: EMS denotes emergency medical service; CPR denotes cardiopulmonary resuscitation. Subgroup analyses adjusted for age, sex, witnessed, bystander CPR, initial rhythm, time from emergency call to drug administration (time from EMS arrival at scene to drug administration for time from call to EMS arrival analysis), etiology of cardiac arrest where applicable. Confidence interval widths have not been adjusted for multiplicity and may not be used in place of hypothesis testing.

**Table 1:** Characteristics of patients at baseline

CHARACTERISTICS	Intraosseous (IO) route (N=3040)	Intravenous (IV) route (N=3042)	Total (N=6086)
*Age mean ± standard deviation - yr*	*67.8 ± 16.3*	*68.3 ± 15.9*	*68.1 ± 16.1*
No. of patients in analysis	2991	2992	5983
**Missing**	49	50	99
*Sex – no. (%)*			
Male	1941 (63.9%)	1951 (64.1%)	3892 (64.0%)
Female	1063 (35.0%)	1048 (34.5%)	2111 (34.7%)
**Missing**	36 (1.2%)	43 (1.4%)	79 (1.3%)
*Location (Utstein style) – no. (%)*			
Home	2392 (78.7%)	2422 (79.6%)	4814 (79.2%)
Industrial/ workplace	54 (1.8%)	47 (1.5%)	101 (1.7%)
Sport/ recreation event	24 (0.8%)	27 (0.9%)	51 (0.8%)
Street/ highway	245 (8.1%)	242 (8.0%)	487 (8.0%)
Public building	99 (3.3%)	96 (3.2%)	195 (3.2%)
Assisted living/nursing home	111 (3.7%)	92 (3.0%)	203 (3.3%)
Education Institution	0 (0.0%)	1 (0.0%)	1 (0.0%)

Others	98 (3.2%)	103 (3.4%)	201 (3.3%)
**Missing**	17 (0.6%)	12 (0.4%)	29 (0.5%)
*Initial cardiac rhythm – no. (%)*			
Shockable	564 (18.6%)	634 (20.8%)	1198 (19.7%)
Ventricular fibrillation	*499 (16.4%)*	*571 (18.8%)*	*1070 (17.6%)*
Pulseless ventricular tachycardia	*12 (0.4%)*	*17 (0.6%)*	*29 (0.5%)*
AED shockable	*53 (1.7%)*	*46 (1.5%)*	*99 (1.6%)*
Non-shockable	2414 (79.4%)	2358 (77.5%)	4772 (78.5%)
Asystole	*1689 (55.6%)*	*1638 (53.8%)*	*3327 (54.7%)*
Pulseless electrical activity	*681 (22.4%)*	*656 (21.6%)*	*1337 (22.0%)*
AED non-shockable	*44 (1.4%)*	*64 (2.1%)*	*108 (1.8%)*
**Missing**	**62 (2.0%)**	**50 (1.6%)**	**112 (1.8%)**
*Initial etiology – no. (%)*			
Medical	2484 (81.7%)	2480 (81.5%)	4964 (81.6%)
Trauma	48 (1.6%)	38 (1.2%)	86 (1.4%)
Drowning	8 (0.3%)	7 (0.2%)	15 (0.2%)
Overdose	57 (1.9%)	67 (2.2%)	124 (2.0%)
Asphyxia	86 (2.8%)	87 (2.9%)	173 (2.8%)
Electrocution	2 (0.1%)	1 (0.0%)	3 (0.0%)
**Missing**	**355 (11.7%)**	**362 (11.9%)**	**717 (11.8%)**
*Witness of cardiac arrest – no. (%)*			
Unwitnessed	1164 (38.3%)	1109 (36.5%)	2273 (37.4%)
EMS witnessed	194 (6.4%)	183 (6.0%)	377 (6.2%)
Bystander witnessed	1645 (54.1%)	1703 (56.0%)	3348 (55.0%)
**Missing**	**37 (1.2%)**	**47 (1.5%)**	**84 (1.4%)**
*Bystander commenced CPR – no. (%)*			
Yes	2089 (68.7%)	2145 (70.5%)	4234 (69.6%)
No	701 (23.1%)	670 (22.0%)	1371 (22.5%)
Not applicable (EMS witnessed)	193 (6.3%)	181 (6.0%)	374 (6.1%)
**Missing**	**6 (0.2%)**	**7 (0.2%)**	**13 (0.2%)**
*Public Access Defibrillator used – no. (%)*			
Yes	251 (8.3%)	238 (7.8%)	489 (8.0%)
No	2638 (86.8%)	2674 (87.9%)	5312 (87.3%)
Not applicable (EMS witnessed)	109 (3.6%)	100 (3.3%)	209 (3.4%)
**Missing**	**42 (1.4%)**	**30 (1.0%)**	**72 (1.2%)**

**Table 2:** Time intervals between key events and cardiac arrest treatment

TIME INTERVALS	Intraosseous (IO) route (N=3040)	Intravenous (IV) route (N=3042)	Total (N=6082)
*Time from emergency call to arrival at scene*			
No. of patients in analysis	3026	3031	6057	
Median (IQR) – min †	8.0 (5.0, 12.0)	8.0 (5.0, 12.0)	8.0 (5.0, 12.0)	
*Time from arrival at scene to gain vascular access*			
No. of patients in analysis	2870	2857	5727	
Median (IQR) – min †	12.0 (9.0, 16.0)	12.0 (9.0, 17.0)	12.0 (9.0, 17.0)	
*Time from arrival at scene to drug administration*			
No. of patients in analysis	2847	2811	5658	
Median (IQR) – min †	14.0 (11.0, 19.0)	15.0 (11.0, 20.0)	14.0 (11.0, 19.0)	
*Time from emergency call to gain vascular access*			
No. of patients in analysis	2874	2867	5741	
Median (IQR) – min †	21.0 (17.0, 27.0)	22.0 (17.0, 28.0)	21.0 (17.0, 27.0)	
*Time from emergency call to drug administration*			
No. of patients in analysis	2857	2826	5683	
Median (IQR) – min †	24.0 (19.0, 30.0)	24.0 (20.0, 31.0)	24.0 (19.0, 30.0)	
*Time from arrival at scene to EMS transport*			
No. of patients in analysis	992	1107	2099	
Median (IQR) – min †	56.0 (42.0, 71.5)	55.0 (43.0, 70.0)	55.0 (42.0, 71.0)	
*Time from emergency call to hospital arrival*			
No. of patients in analysis	1009	1121	2130
Median (IQR) – min †	78.0 (62.0, 99.0)	78.0 (64.0, 97.0)	78.0 (63.0, 98.0)
*Site of first successful vascular access*			
Intraosseous access – no (%)*	2871 (94.4%)	992 (32.6%)	3863 (63.5%)
*Proximal humerus*	519 (17.1%)	160 (5.3%)	679 (11.2%)
*Proximal tibial*	2233 (73.5%)	780 (25.6%)	3013 (49.5%)
*Other*	119 (3.9%)	52 (1.7%)	171 (2.8%)
Intravenous access – no (%)*	107 (3.5%)	1964 (64.6%)	2071 (34.1%)
*Central*	3 (0.1%)	41 (1.3%)	44 (0.7%)
*Peripheral*	99 (3.3%)	1857 (61.0%)	1956 (32.2%)
*Other*	5 (0.2%)	66 (2.2%)	71 (1.2%)
*Epinephrine*			
Administered- *– no. (%)*	2866 (94.3%)	2836 (93.2%)	5702 (93.8%)
Dose (mg)- median (IQR)	5.0 (3.1, 8.0)	5.0 (3.0, 8.0)	5.0 (3.0, 8.0)
Amiodarone administered*– no. (%)*	480 (15.8%)	524 (17.2%)	1004 (16.5%)
*Number of defibrillator shocks- median (IQR)*	3.0 (1.0, 6.0)	3.0 (1.0, 6.0)	3.0 (1.0, 6.0)
*Supraglottic airway – no. (%)*			
Yes	2765 (91.0%)	2747 (90.3%)	5512 (90.6%)
No	220 (7.2%)	237 (7.8%)	457 (7.5%)
*Tracheal tube – no. (%)*			
Yes	648 (21.3%)	613 (20.2%)	1261 (20.7%)
No	2318 (76.3%)	2364 (77.7%)	4682 (77.0%)
Transported to hospital – no. (%)			
Yes	1024 (33.7%)	1136 (37.3%)	2160 (35.5%)
No	2016 (66.3%)	1906 (62.7%)	3922 (64.5%)

† Among cardiac arrests that were witnessed by paramedics, the interval between the emergency call and the cardiac arrest event was taken as 0 minutes.

* Of the 107 patients in the intraosseous group that received intravenous access as their first successful vascular access, 88 were categorized as a crossover. Of the 992 patients in the intravenous group that received intraosseous access as their first successful vascular access, 369 were categorized as a crossover. Crossover is defined as the use of the non-randomized drug route prior to two unsuccessful attempts at the randomized route.

**Table 3:** Primary and Secondary Outcomes

OUTCOME	Intraosseous (IO) route	Intravenous (IV) route	Risk/mean difference (95% CI) †		Odds/hazard/incidence rate Ratio (95% CI) †	
			Unadjusted	Adjusted‡	Unadjusted	Adjusted
*Primary outcome*						
Survival at 30 days – no./total no. (%)	137/3030 (4.5%)	155/3034 (5.1%)	-0.6% (-1.7%, 0.5%)	-0.2% (-1.1%, 0.8%)	0.880 (0.695, 1.113)	0.945 (0.676, 1.322), p=0.741
*Secondary outcomes*						
Return to spontaneous circulation (ROSC) anytime – no./total no. (%)	1092/3031 (36.0%)	1186/3035 (39.1%)	-3.0% (-5.5%, -0.6%)	-3.2% (-5.9%, - 0.6%)	0.878 (0.791, 0.974)	0.863 (0.765, 0.974)
Time to return of spontaneous circulation (ROSC)– mins- median (IQR)	33 (24.0, 43.0)	32 (24.0, 43.0)	0.757 (-1.062, 2.576)	0.451 (-0.818, 1.719)	0.896 (0.823, 0.975)^Δ^	0.889 (0.808, 0.979)^Δ^
Sustained return to spontaneous circulation (ROSC) at hospital handover – no./total no. (%)	654/3016 (21.7%)	744/3023 (24.6%)	-2.9% (-5.1%, -0.8%)	-2.6% (-4.8%, - 0.3%)	0.848 (0.752, 0.956)	0.853 (0.741, 0.983)
Survival to hospital discharge - no./total no. (%)	112/3012 (3.7%)	120/3012 (4.0%)	-0.3% (-1.2%, 0.7%)	0.0% (-0.9%, 0.8%)	0.931 (0.716, 1.210)	0.996 (0.679, 1.461)
Length of hospital stay– days- median (IQR)						
Patients who survived	18.0 (11, 32)	16.5 (7, 31)	3.122 (-4.698, 10.942)	7.681 (-4.392, 19.754)	-	-
Patients who died	0.0 (0, 0)	0.0 (0, 0)	-0.229 (-0.483, 0.024)	-0.178 (-0.454, 0.098)	-	-
Favourable Neurological						
Outcome at Hospital Discharge: Modified Rankin Scale at Discharge – no./total no. (%)						
(0-3) favourable outcome	80 (2.7%)	85 (2.8%)				
(4-6) unfavourable outcome	2914 (97.3%)	2901 (97.2%)	-0.2% (-1.0%, 0.7%)	-0.1% (-0.8%, 0.6%)	0.937 (0.687, 1.277)	0.914 (0.567, 1.474)
Adverse event (per 1000 patients)	1/3040 (0.33)	0/3042 (0)		-	1.003 (0.856, 1.176), p=0.968	-
Serious adverse event (per 1000 patients)	0/3040 (0)	0/3042 (0)	-	-	-	-

IQR-Interquartile range

† the risk difference (post-hoc test), hazard ratio (HR), incidence rate ratio (IRR) or mean difference are for IO versus IV. Risk difference are reported since odds ratios may over-estimate the magnitude of treatment effect. Treatment differences are adjusted for: age, sex, witness status (EMS versus bystander), bystander CPR (yes/no), initial rhythm (shockable versus non-shockable), time from emergency call to drug administration, etiology of cardiac arrest (medical versus non-medical). Risk of adverse event is assessed using Poisson regression and IRR is reported. No comparison is conducted for serious adverse event. Confidence interval widths have not been adjusted for multiplicity and may not be used in place of hypothesis testing.

‡ Adjusted risk difference was estimated using SAS macro Margins (https://support.sas.com/kb/63/038.html).

Δ Cause-specific hazard function was used to estimate the hazards of ROSC. Death before any ROSC is considered as a competing risk. Proportional hazard assumption was not violated for both unadjusted and adjusted analyses.

Modified Rankin Score is assessed on a 7-point scale from 0 to 6, namely: 0- No symptoms, 1- No significant disability, 2- Slight disability, 3- Moderate disability, 4- Moderate severe disability, 5- Severe disability, 6- Dead. A score of 0-3 is categorized as a favorable neurological outcome.^19^

## References

[1] Perkins GD, Ji C, Deakin CD (2018). A Randomized Trial of Epinephrine in Out-of-Hospital Cardiac Arrest. New England Journal of Medicine.

[2] Kudenchuk PJ, Brown SP, Daya M (2016). Amiodarone, Lidocaine, or Placebo in Out-of-Hospital Cardiac Arrest. New England Journal of Medicine.

[3] Perkins GD, Kenna C, Ji C (2020). The influence of time to adrenaline administration in the Paramedic 2 randomised controlled trial. Intensive Care Medicine.

[4] Rahimi M, Dorian P, Cheskes S, Lebovic G, Lin S (2022). Effect of Time to Treatment With Antiarrhythmic Drugs on Return of Spontaneous Circulation in Shock-Refractory Out-of-Hospital Cardiac Arrest. Journal of the American Heart Association.

[5] Vallentin MF, Granfeldt A, Meilandt C (2021). Effect of Intravenous or Intraosseous Calcium vs Saline on Return of Spontaneous Circulation in Adults With Out-of-Hospital Cardiac Arrest: A Randomized Clinical Trial. JAMA.

[6] Reades R, Studnek JR, Vandeventer S, Garrett J (2011). Intraosseous Versus Intravenous Vascular Access During Out-of-Hospital Cardiac Arrest: A Randomized Controlled Trial. Annals of Emergency Medicine.

[7] Ross EM, Mapp J, Kharod C, Wampler DA, Velasquez C, Miramontes DA (2016). Time to epinephrine in out-of-hospital cardiac arrest: A retrospective analysis of intraosseous versus intravenous access. American Journal of Disaster Medicine.

[8] Granfeldt A, Avis SR, Lind PC (2020). Intravenous vs. intraosseous administration of drugs during cardiac arrest: A systematic review. Resuscitation.

[9] Hooper A, Nolan JP, Rees N, Walker A, Perkins GD, Couper K (2022). Drug routes in out-of-hospital cardiac arrest: A summary of current evidence. Resuscitation.

[10] Hsieh Y-L, Wu M-C, Wolfshohl J (2021). Intraosseous versus intravenous vascular access during cardiopulmonary resuscitation for out-of-hospital cardiac arrest: a systematic review and meta-analysis of observational studies. Scandinavian Journal of Trauma, Resuscitation and Emergency Medicine.

[11] Soar J, Böttiger BW, Carli P (2021). European Resuscitation Council Guidelines 2021: Adult advanced life support. Resuscitation.

[12] Panchal AR, Bartos JA, Cabañas JG (2020). Part 3: Adult Basic and Advanced Life Support: 2020 American Heart Association Guidelines for Cardiopulmonary Resuscitation and Emergency Cardiovascular Care. Circulation.

[13] Brebner C, Asamoah-Boaheng M, Zaidel B (2024). The association of intravenous vs. humeral-intraosseous vascular access with patient outcomes in adult out-of-hospital cardiac arrests. Resuscitation.

[14] Agostinucci J-M, Alhéritière A, Metzger J (2024). Evolution of the use of intraosseous vascular access in prehospital advanced cardiopulmonary resuscitation: The IOVA-CPR study. International Journal of Nursing Practice.

[15] Vadeyar S, Buckle A, Hooper A (2023). Trends in use of intraosseous and intravenous access in out-of-hospital cardiac arrest across English ambulance services: A registry-based, cohort study. Resuscitation.

[16] Soar J, Berg KM, Andersen LW (2020). Adult Advanced Life Support: 2020 International Consensus on Cardiopulmonary Resuscitation and Emergency Cardiovascular Care Science with Treatment Recommendations. Resuscitation.

[17] Couper K, Ji C, Lall R (2024). Route of drug administration in out-of-hospital cardiac arrest: A protocol for a randomised controlled trial (PARAMEDIC-3. Resusc Plus.

[18] Perkins GD, Jacobs IG, Nadkarni VM (2015). Cardiac Arrest and Cardiopulmonary Resuscitation Outcome Reports: Update of the Utstein Resuscitation Registry Templates for Out-of-Hospital Cardiac Arrest. Resuscitation.

[19] Haywood K, Whitehead L, Nadkarni VM (2018). COSCA (Core Outcome Set for Cardiac Arrest) in Adults: An Advisory Statement From the International Liaison Committee on Resuscitation. Resuscitation.

[20] O’Brien PC, Fleming TR (1979). A multiple testing procedure for clinical trials. Biometrics.

[21] McCoy CE (2017). Understanding the Intention-to-treat Principle in Randomized Controlled Trials. West J Emerg Med.

[22] European Medicines Agency ICH E9 (R1) addendum on estimands and sensitivity analysis in clinical trials to the guideline on statistical principles for clinical trials Step 5.

[23] Rimawi M, Hilsenbeck SG (2012). Making sense of clinical trial data: is inverse probability of censoring weighted analysis the answer to crossover bias?. J Clin Oncol.

[24] Sui Y, Bu X, Duan Y, Li Y, Wang X (2023). https://www.pharmasug.org/proceedings/2023/SD/PharmaSUG-2023-SD-069.pdf.

[25] Khan MS, Ochani RK, Shaikh A (2019). Fragility Index in Cardiovascular Randomized Controlled Trials. Circulation: Cardiovascular Quality and Outcomes.

[26] Perkins GD, Neumar R, Hsu CH (2024). Improving Outcomes After Post-Cardiac Arrest Brain Injury: A Scientific Statement From the International Liaison Committee on Resuscitation. Resuscitation.

[27] Berger D, Petrie A, Lubin JS (2023). The Ability of Paramedics to Accurately Locate Correct Anatomical Sites for Intraosseous Needle Insertion. Cureus.

[28] Daya MR, Leroux BG, Dorian P (2020). Survival After Intravenous Versus Intraosseous Amiodarone, Lidocaine, or Placebo in Out-of-Hospital Shock-Refractory Cardiac Arrest. Circulation.

[29] Ko Y-C, Lin H-Y, Huang EP-C (2024). Intraosseous versus intravenous vascular access in upper extremity among adults with out-of-hospital cardiac arrest: cluster randomised clinical trial (VICTOR trial). BMJ.

[30] Nishiyama C, Kiguchi T, Okubo M (2023). Three-year trends in out-of-hospital cardiac arrest across the world: Second report from the International Liaison Committee on Resuscitation (ILCOR). Resuscitation.

[31] Nolan JP, Sandroni C, Böttiger BW (2021). European Resuscitation Council and European Society of Intensive Care Medicine guidelines 2021: post-resuscitation care. Intensive Care Medicine.

[32] Meilandt C, Fink Vallentin M, Blumensaadt Winther K (2023). Intravenous vs. intraosseous vascular access during out-of-hospital cardiac arrest – protocol for a randomised clinical trial. Resuscitation Plus.

