# Supplementary material for: A Randomized Trial of Drug Route in Out-of-Hospital Cardiac Arrest
Source: N Engl J Med. Author manuscript; Available in PMC 2024 Nov 1. (PMC7616768; doi:10.1056/NEJMoa2407780)
Supplement: supplement [file EMS199114-supplement-supplement.pdf]

A randomized trial of drug route in out-of-hospital cardiac arrest

Supplementary Appendix

Table of Contents

Section one: Trial Investigators/ collaborators and committees 3
Trial investigators and collaborators 3
Committees 5
Trial methods 6
System description 6
Resuscitation Guidelines in the UK 8
Deferred consent model 10
Patient and public involvement (PPI) 11
National data opt-out 13
Representativeness of study participants 14
Co-enrolment with other research studies 15
Supplemental figures 16
Figure S1: Kaplan Meier curve for survival at 30 days by treatment arm 16
Figure S2: Cumulative incidence curve for time to first ROSC by treatment arm 17
Figure S3: Cumulative incidence curve for time to sustained ROSC at hospital handover by treatment arm 18
Figure S4: Kaplan Meier curve for survival to hospital discharge by treatment arm 19
Figure S5: Summary of unadjusted subgroup analyses for primary outcome 20
Figure S6: Survival with a Favourable Neurological Outcome at Hospital Discharge (Modified Rankin Score) 21
Figure S7: Tipping point analysis of survival at 30 days 22
Figure S8: Rate of survival at 30 days over the recruitment period by treatment arm 23
Figure S9: Rate of return of spontaneous circulation at any time over the recruitment period by treatment arm 24
Figure S10: Rate of first attempt success at vascular access over the recruitment period by treatment arm 25
Supplemental tables 26
Table S1: Overview of participating centres and recruitment 26
Table S2: Screening log reasons for non-randomization by site 27

Table S3: Participant ethnicity 28
Table S4: Patient status from prior to hospitalization to the end of study 29
Table S5: Randomization balance by emergency medical service 30
Table S6: Protocol deviations, violations, and withdrawal by treatment arm 31
Table S7: Non-compliance by treatment arm 32
Table S8: Time from emergency medical service arrival to vascular access by rhythm type 34
Table S9: Vascular access success rates 35
Table S10: Fragility Index of survival at 30 days (Sensitivity analysis) 36
Table S11: Analysis of imputed survival at 30 days and functional outcomes (Sensitivity analysis) 37
Table S12: Inverse probability of censoring weighted analysis of survival at 30 days (sensitivity analysis) 38
Table S13: Adjusted outcome analyses using alternative time covariates (post-hoc sensitivity analyses) 39
Table S14: Summary of Adverse Events 41
Table S15: List of Adverse Events by treatment arm 41
Table S16: List of serious Adverse Events by treatment arm 41
References 42

Section one: Trial Investigators/ collaborators and committees

Trial investigators and collaborators

This section lists personnel who contributed sufficiently to PARAMEDIC-3 activities at participating centres and the trial co-ordinating centre (Warwick Clinical Trials Unit). Participating centre lists include principal investigators and lead research paramedics, some of whom are listed as manuscript authors. The list for the University of Warwick excludes individuals that are also listed as authors. Some collaborators have moved institution since working on PARAMEDIC-3, but are listed at the site or institution where they contributed to PARAMEDIC-3.

Devon Air Ambulance; Exeter; UK

Mr Nigel Lang M.Sc; Dr Belinda Tibbetts Ph.D; Mrs Hannah Trebilcock

East of England Ambulance Service NHS Trust; Cambridge; UK

Mrs Theresa Foster M.Sc.; Mrs Shona Brown B.Sc.; Mr Callum Brown B.Sc.; Mrs Emma Duncan B.Sc.; Mrs Tessa Nokes M.Sc.; Ms Larissa Prothero Ph.D. ;

East Midlands Ambulance Service NHS Trust; Nottingham; UK

Mr Robert E.S. Spaight MMEDSci; Miss Gemma L. Squires B.Sc.; Dr Gregory A. Whitley Ph.D.; Miss Meera Chauhan BA; Miss Rachel Clarke MA; Mrs Dorothy Hutchinson M.Sc. .

London Ambulance Service NHS Trust; London; UK

Prof Rachael Fothergill Ph.D; Mr Justin Kearney M.Sc.; Mr Jakob Falloon M.Sc.; Mr Adam Mellett-Smith M.Sc.; Mr Gabriel Palti B.Sc.; Mr Martim Rodrigues B.Sc.; Miss Caitlin Sears B.Sc.

North East Ambulance Service NHS Foundation Trust; Newcastle Upon Tyne; UK

Mr Karl Charlton MRES; Ms Emma Burrow B.A.; Mr Matthew Limmer B.Sc.; Ms Sarah Hepburn; Mrs Michelle Jackson

North West Ambulance Service NHS Trust; Bolton; UK

Mr Steve Bell M.Sc.; Mrs Michelle Waddington DipHE; Mr Adam Wright M.Sc.; Ms Sandra Igbodo M.Sc.; Mr Jesse Oliver; Mrs Betty Pennington† M.Sc.

South Central Ambulance Service NHS Foundation Trust; Bicester; UK

Prof Charles D. Deakin M.D.; Mrs Martina Brown M.Sc.; Mr Andrew Claxton Dip HE; Ms Victoria Dra'gon B.Sc.; Dr John Black FRCSEd FIMCRCSEd FRCEM; Mr Chris Brooker DIP HE; Mr Ben Keeble BA(Hons); DIP Para; Miss Siobhan Law PGcert; Mr Rob MacKenzie MCPara; Ms Sue McSheaffrey B.Sc.(Hons); Mr Jonathan Phillips B.Sc.(Hons); Dr Helen Pocock Ph.D.; Miss Melody Pooles FdSc ; Mrs Lisa Pringle B.Sc.; Heather Smith B.Sc. (Hons); Mr David Worsfold MStJ FdSc

South East Coast Ambulance Service NHS Foundation Trust; Crawley; UK

Prof Julia Williams Ph.D.; Gemma Bradley FdSc; Mr Craig Mortimer M.Sc.; Mr Peter Eaton-Williams M.Sc.

South Western Ambulance Service NHS Foundation Trust; Exeter; UK

Miss Ria Osborne M.BioMedSc; Mrs Sarah Wiltshire B.Sc.; Dr Sarah Black Ph.D.; Mrs Alison Coppola M.Sc.

Welsh Ambulance Services University NHS Trust; Cwmbran; Wales; UK

Prof Nigel Rees Ph.D.; Mrs Carla Mills B.Sc. Mrs Charlotte Evans-Waugh; Mr Dmitri Holloway; Mrs Lauren Smyth M.Sc.; Mrs Lauren Williams B.Sc. (Hons); Mrs Cendl Xanthe M.Sc

West Midlands Ambulance Service University NHS Foundation Trust; Brierley Hill; UK

Mr Jason V. Wiles DipHE; Mrs Christine Evans B.Sc; Mr Joshua Miller M.B.A.; Mrs Rhiannon Boldy; Ms Zoe Green; Ms Imogen Gunson M.Sc.; Mr Paul Lockley; Mr Robert Millard; Mr Andy Rosser M.Sc.; Mr Owen Stanley; Mr Brian Davidson M.Sc; Dr Alison Walker M.B., B.Chir.

University of Warwick; Coventry; UK

Mr Scott Regan B.A.; Ms Emma Skilton; Ms Chloe Norman M.Res.; Ms Loraine Chowdhury; Miss Laurilee Sprauve; Ms Fatimah J. Chowdhury; Dr Hannah Noordali Ph.D; Ms Jeskaran Rai; Ms Natalie Strickland; Ms Claire Daffern; Mrs Jill Wood; Mrs Emily Long; Dr Rebecca Kandiyali

† - Deceased

Committees

Data monitoring and ethics committee

The DMeC comprised four individual including Prof Marion Campbell (Chair), Prof Kathy Rowan and Dr Jasmeet Soar

Trial steering committee

Prof Steve Goodacre (Chair), Prof Helen Snooks, Prof Jonathan Wyllie, Mr Steve Irving, Mr David Bywater, Professor Julia Williams, Mrs Anne Devrell*, Mr Elyas Khalifa*, Prof Ly-Mee Yu, Prof Gavin Perkins

*- Devrell and Khalifa were patient and public involvement representatives

Trial management group

Prof Gavin Perkins, Dr Keith Couper, Prof Ranjit Lall, Dr Chen Ji, Prof Charles Deakin, Prof Rachael Fothergill, Prof Jerry Nolan, Mr John Long*, Prof James Mason, Mr Felix Michelet, Dr Henry Nwankwo, Prof Tom Quinn, Prof Anne-Marie Slowther, Dr Michael Smyth, Dr Alison Walker, Mr Scott Regan, Ms Emma Skilton, Ms Chloe Norman, Ms Kath Starr, Dr Sara Wood, Ms Loraine Chowdhury.

*- Long was a patient and public involvement representatives

Patient advisory group

The patient advisory group was chaired by Mr John Long and comprised six members, including: Neil Davidson, Mr Ben Thom-Wood, Mr Graham Howkins, Marion Thompson, and Susan Jenkins

Trial methods

System description

The United Kingdom is served by 13 National Health Service ambulance services. Three of these services serve an entire country (Northern Ireland, Scotland, Wales). England is served by 10 regional ambulance services.

The UK is also served by 21 air ambulance charities. These charities provide an enhanced clinical response, led by either specialist critical care paramedics or physicians. These specialist teams may travel to an incident by helicopter or car, depending on weather, time of day, helicopter availability, and location of the incident. Typically, air ambulance charities are dispatched to attend incidents based on specific call criteria (e.g. major trauma) or the request of the ambulance personnel on scene.

In the event of a medical emergency in the UK requiring an ambulance, the caller will phone a national number (999/ 112) which automatically allocates the caller to the ambulance service that covers that geographical area. Cardiac arrest calls are allocated the highest priority response with a target mean response time of 7-minutes. The resources allocated to a cardiac arrest vary by service, based on organizational policy and resource availability. This may include community first responders, ambulances, rapid response cars, and air ambulances. Across all services, the minimum response to a cardiac arrest would be a single ambulance, of which one crew member would be a paramedic.

Paramedics in the UK are trained and competent in delivering advanced life support, including manual defibrillation, insertion of advanced airways (tracheal intubation/ supraglottic airway depending on service), vascular access (intravenous/ intraosseous access), and drug therapy (epinephrine, amiodarone, intravenous fluids). Paramedics may be supported by an Emergency Medical Technician or Emergency Care Assistant who are competent in delivering basic life support and may, depending on skill-level and service, be able to deliver some enhanced skills such as supraglottic airway insertion.

On arrival at a cardiac arrest, responders will determine the appropriateness of continuing or commencing resuscitation, based on national criteria (e.g. unequivocal signs of death, do not attempt cardiopulmonary resuscitation order). Once started, paramedics may terminate resuscitation attempts in accordance with national guidelines.[1]

Across England and Wales, there are approximately 30,000 cardiac arrests treated by NHS ambulance services each year.[2] The overall rate for survival at hospital discharge is 7.9%.

Participants in the PARAMEDIC-3 trial were recruited from nine English NHS ambulance services, the Welsh NHS ambulance service, and one standalone air ambulance charity. Joint governance arrangement in one NHS ambulance service facilitated recruitment both within the ambulance service and its local air ambulance service.

The Scottish and Northern Irish ambulance services were unable to participate due to differences in legislation covering the recruitment of research participants without prior consent in those regions. One English ambulance service was unable to participate in the trial due to capacity issues.

Our map of the mainland UK shows the geographical areas in green covered by ambulance services that recruited to the PARAMEDIC-3 trial. The green area covers a wide mix of urban and rural areas.

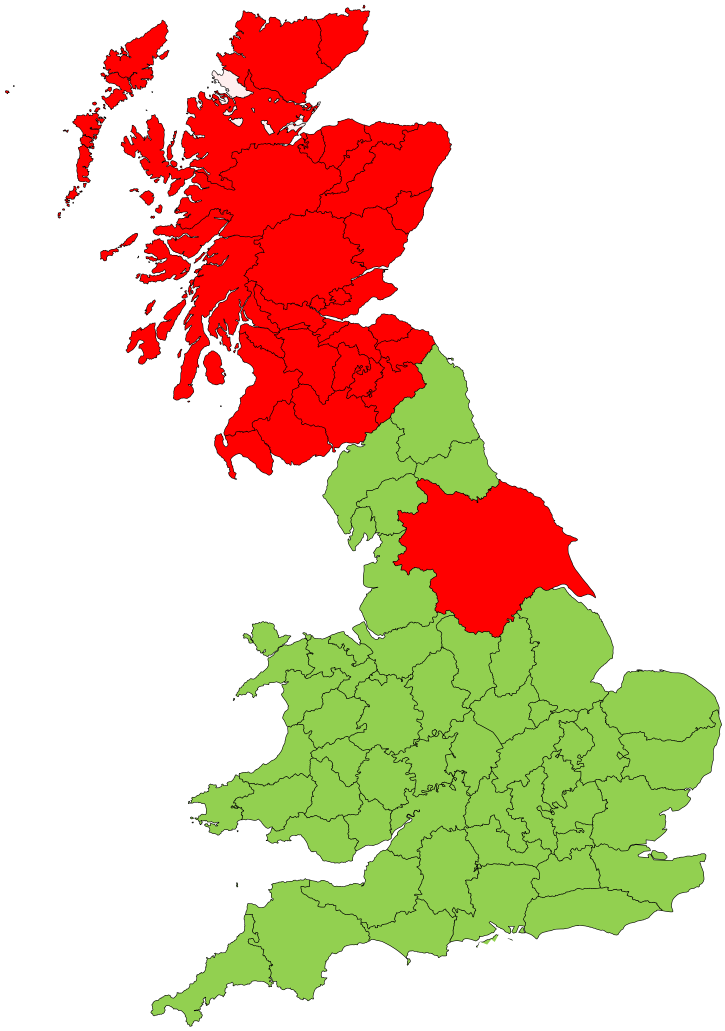

All organizations participating in the PARAMEDIC-3 trial used the Teleflex Arrow EZ-IO system for securing intraosseous access (a drill-based system). The trial did not protocolize use of a specific device or system for securing intraosseous access, such that each organization continued to use the system that was in use prior to the trial starting. The decision on what device or system to use for intraosseous access is determined locally based on factors such as availability and user experience. Over recent years, use of intraosseous access in the UK pre-hospital system for adult cardiac arrest in routine care has increased from 22.8% patients in 2015 to 42.5% patients in 2020.[3]

Resuscitation Guidelines in the UK

Cardiac arrest management in UK ambulance services is based on the guidelines developed by Resuscitation Council UK. These guidelines are informed by the European Resuscitation Council guidelines and International Liaison Committee on Resuscitation treatment recommendations.[4,5] The 2021 Resuscitation Council UK Adult Advanced Life Support algorithm is shown below.

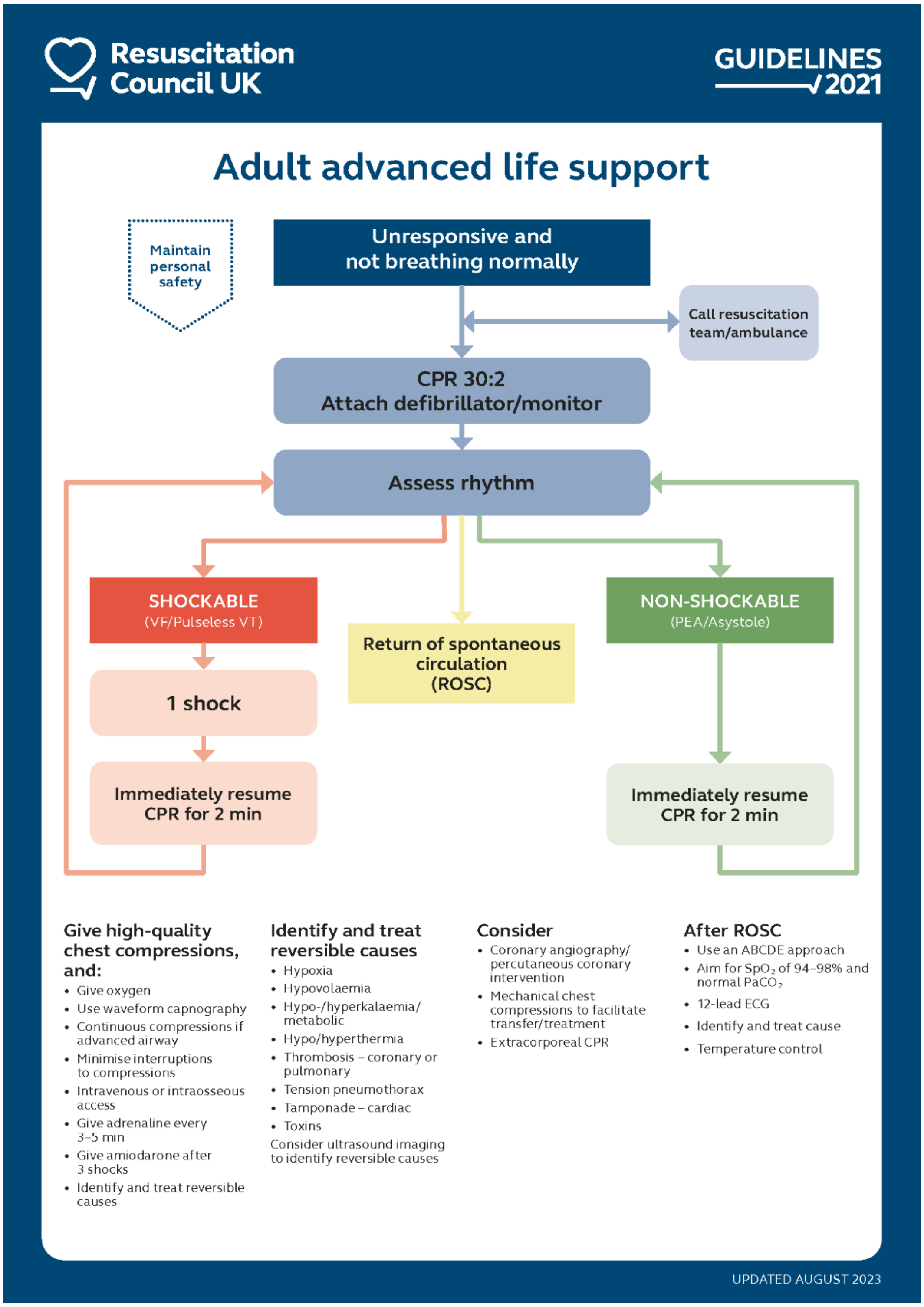

Reproduced with kind permission of Resuscitation Council UK

The most common drug used in adult cardiac arrest is epinephrine (adrenaline). These guidelines recommend that epinephrine (1mg) is given immediately after commencement of cardiopulmonary resuscitation for non-shockable rhythms (pulseless electrical activity/ asystole) and following the third defibrillation attempt for shockable rhythms (ventricular fibrillation/ ventricular tachycardia). Once epinephrine has been given, the dose is repeated every 3-5 minutes (every two cycles).

Deferred consent model

PARAMEDIC-3 was approved by a research ethics committee to recruit participants under a deferred consent model in accordance with English and Welsh legislation. The Health Research Authority in the UK has developed a framework to support the design and review of research in the emergency care setting that seeks to rely on such a deferred consent model.[6] The framework includes considerations such as the need for the research, the need to recruit participants who lack mental capacity, the urgency of treatment, and the feasibility of consultation with a professional or personal consultee prior to enrolment. In our protocol, we detail how these considerations applied to PARAMEDIC-3. PARAMEDIC-3 recruited participants in cardiac arrest who required time-critical treatment, such that any delay to consult a representative of the patient would be harmful to the patient. The interventions being compared (intraosseous/ intravenous vascular access) were already in routine UK clinical practice, with evidence showing that the use of intraosseous access was increasing over time.[3] The international resuscitation community had highlighted uncertainty as to the optimum vascular access strategy in cardiac arrest and highlighted the urgent need for a randomized clinical trial.[4] Recruitment was undertaken by registered health care professionals, including doctors, nurses, and paramedics (collectively referred to throughout the paper as paramedics as most recruitment was led by paramedics). The protocol stated that where the healthcare professional assessing eligibility determined that a specific strategy was in the best interests of the patient, then that individual should not be enrolled.

Following cardiac arrest, consent was sought from surviving patients or, if the patient lacked mental capacity, from an appropriate consultee. The focus of this consent decision was the collection of patient-reported outcome measures at 3-months and 6-months. Patient information sheets were translated into seven commonly used languages: Bengali, French, Polish, Portuguese, Punjabi, Urdu, Welsh.

Our approach was supported by trial patient and public representatives.

Patient and public involvement (PPI)

The following description of patient and public involvement in PARAMEDIC-3 is based on the GRIPP2 short-form reporting checklist of patient and public involvement in research.[7]

Aim

In PARAMEDIC-3, we sought to develop a strategy to enable patients and members of the public to make a meaningful contribution to the design, delivery, and dissemination of the trial.

Methods

We engaged with patients and members of the public in PARAMEDIC-3 in three ways:

1) A patient and public representative (John Long) was a trial co-applicant. Long sat as a full member of the trial management group and chaired the patient advisory group. Long undertook a similar role for both PARAMEDIC (mechanical CPR in out-of-hospital cardiac arrest) and PARAMEDIC-2 (epinephrine for out-of-hospital cardiac arrest).[8,9]
2) Two patient and public representatives sat as independent members of the trial steering committee.
3) Patient advisory panel: we convened a patient advisory panel comprised of six individuals. We planned for the advisory panel to meet approximately every six-months. We identified panel members through investigator networks and advertisement through local patient and public involvement groups. We sought to recruit a diverse group (gender, ethnicity, personal experience of critical illness/ cardiac arrest). The group was convened to provide advice to the trial management group in relation to key aspects of the trial design, delivery, and dissemination.

All patient and public contributors were recompensed for their time on an hourly basis at a rate consistent with UK national guidance.

Study and results

Between April 2021 and July 2024, we convened 35 Trial Management Group meetings, of which Long attended 32 (91%) meetings.

To date (August 2024), we have held four Trial Steering Committee meetings. All meetings were attended by at least one patient and public representative and one meeting was attended by both patient and public representatives.

We recruited six individuals to the patient advisory panel. Our membership included three females; one individual from a non-white background and three individuals with personal experience of surviving critical illness, of which two were cardiac arrest survivors. The group met on seven occasions between April 2021 and July 2024. The mean number of attendees per meeting was four (excluding Long). One meeting was attended by patient advisory panel members

from another trial ("Prehospital optimal shock energy for defibrillation (POSED): A cluster randomised controlled feasibility trial") to discuss strategies for co-enrolment between the trials.[10] Each meeting began with introductions and an update on trial progress before focussing on specific issues for which the trial investigators sought advice. There was an opportunity at each meeting for group members to raise any other issues that they wanted to discuss. Key issues discussed at meetings included:

- Advice on the appropriateness of co-enrolment with other trials in relation to acceptability and patient burden.
- Advice on the acceptability of a deferred consent model to inform and support our research ethics application.
- Advice on the acceptability of collecting and processing personally identifiable information without consent to inform and support our research ethics application, including the application of the national data opt-out.
- Review and advice on key patient-facing documents.
- Advice on strategies to disseminate trial findings.

Discussion and conclusions

In PARAMEDIC-3, our patient and public involvement strategy was informed by the approach that we adopted in our PARAMEDIC-2 trial (epinephrine in out-of-hospital cardiac arrest).[8] The strategy effectively supported our objective for the design, delivery and dissemination of the trial to be informed by the perspectives of a range of patients and members of the public.

Our discussions with patients and members of the public provided important insights that informed our approach to a wide range of issues in the trial.

Our patient and public involvement strategy required administrative time to co-ordinate meetings. Overall, we achieved high levels of engagement. Nevertheless, for our patient advisory group, we often found it challenging to identify a meeting time that all individuals could attend. In establishing our patient advisory panel, we used a range of networks to identify potential members, but despite our best efforts, we struggled to identify individuals from a non-white background to join the panel.

Reflections/ critical perspective

Our key learning points from PARAMEDIC-3 are:

- Patient advisory panels provide an effective strategy to elicit the perspectives of a range of patients and members of the public,
- We identified challenges in identifying a diverse range of individuals to the panel and in co-ordinating meetings particularly as some panel members often had competing work commitments,
- The optimum number of individuals to serve on a patient advisory panel is uncertain.

National data opt-out

During the PARAMEDIC-3 trial, the National Health Service implemented the national data opt-out system. This system provides individuals with the opportunity to opt-out of their personally identifiable information being used for research and planning services. Approximately 5% of the population have chosen to sign up to the national data opt-out.

In PARAMEDIC-3, where the participating ambulance service identified a participant as having signed up to the national data opt-out, we adopted the following process:

- In participants that died before consent could be obtained or where the participant could not be contacted to seek consent, we were only to collect and process an anonymized data set. This meant that we were unable to link data with national datasets and we could not collect some data (e.g. extremes of age) where there was a risk that this might be identifiable.
- In patients who survived and who could be contacted, we used the same approach adopted for patients that had not signed up to the national data opt-out. Consent overrode the national data opt-out decision.

Representativeness of study participants

This summary of the representativeness of study participants is reported in line with the NEJM editorial on "Striving for Diversity in Research Studies."[11]

**Category**	**Information**
Disease, problem, or condition under investigation	Out-of-hospital cardiac arrest
Special considerations related to	
Sex and gender	Out-of-hospital cardiac arrest affects predominantly men in a ratio of approximately 2:1.
Age	Out-of-hospital cardiac arrest incidence increases with age with the average age of cardiac arrest patients typically being about 70 years old.
Race or ethnic group	Out-of-hospital cardiac arrest disproportionately affects individuals from a non-white background, both in terms of higher incidence and poorer outcomes.
Geography	The incidence of out-of-hospital cardiac arrest varies markedly across the world. This variability is challenging to interpret as it is influenced by emergency medical service protocols on decisions relating to the commencement of resuscitation, particularly the circumstances in which resuscitation can be withheld.<br><br>In low- and middle-income countries, there are few robust epidemiological studies of out-of-hospital cardiac arrest.
Other considerations	Individuals that live in socially deprived communities are at increased risk of out-of-hospital cardiac arrest and experience poorer outcomes.
Overall representativeness of this trial	The participants in PARAMEDIC-3 were representative of the sex and age of individuals that suffer out-of-hospital cardiac arrest.<br><br>The trial recruited across a wide range of geographical areas in England and Wales, representing a wide range of social deprivation and rurality.<br><br>Ethnicity in PARAMEDIC-3 was prone to substantial missingness as it was predominantly collected through routine health records. These data are collected routinely by the National Health Service, but recent reports have identified important issues in relation to how ethnicity is determined.[12] Nevertheless, after accounting for missingness, the proportion of individuals in each ethnic group is broadly consistent with the 2021 national census.

Co-enrolment with other research studies

PARAMEDIC-3 agreed co-enrolment with other research trials in accordance with UK national guidelines, following review by the trial management group.[13,14] The patient advisory group supported this approach.

Key pre-hospital cardiac arrest studies where there was co-enrolment are summarized in the table below.

Study name	Interventional/ observational	Study identifier	Number of co-enrolled patients
ARREST	Interventional	ISRCTN96585404	18
CABARET	Interventional	NCT05917717	1
POSED	Interventional	ISRCTN16327029	12
RAPID-MIRACLE	Observational	NCT05185063	47
SUB30	Interventional	NCT03700125	2

Supplemental figures

Figure S1: Kaplan Meier curve for survival at 30 days by treatment arm

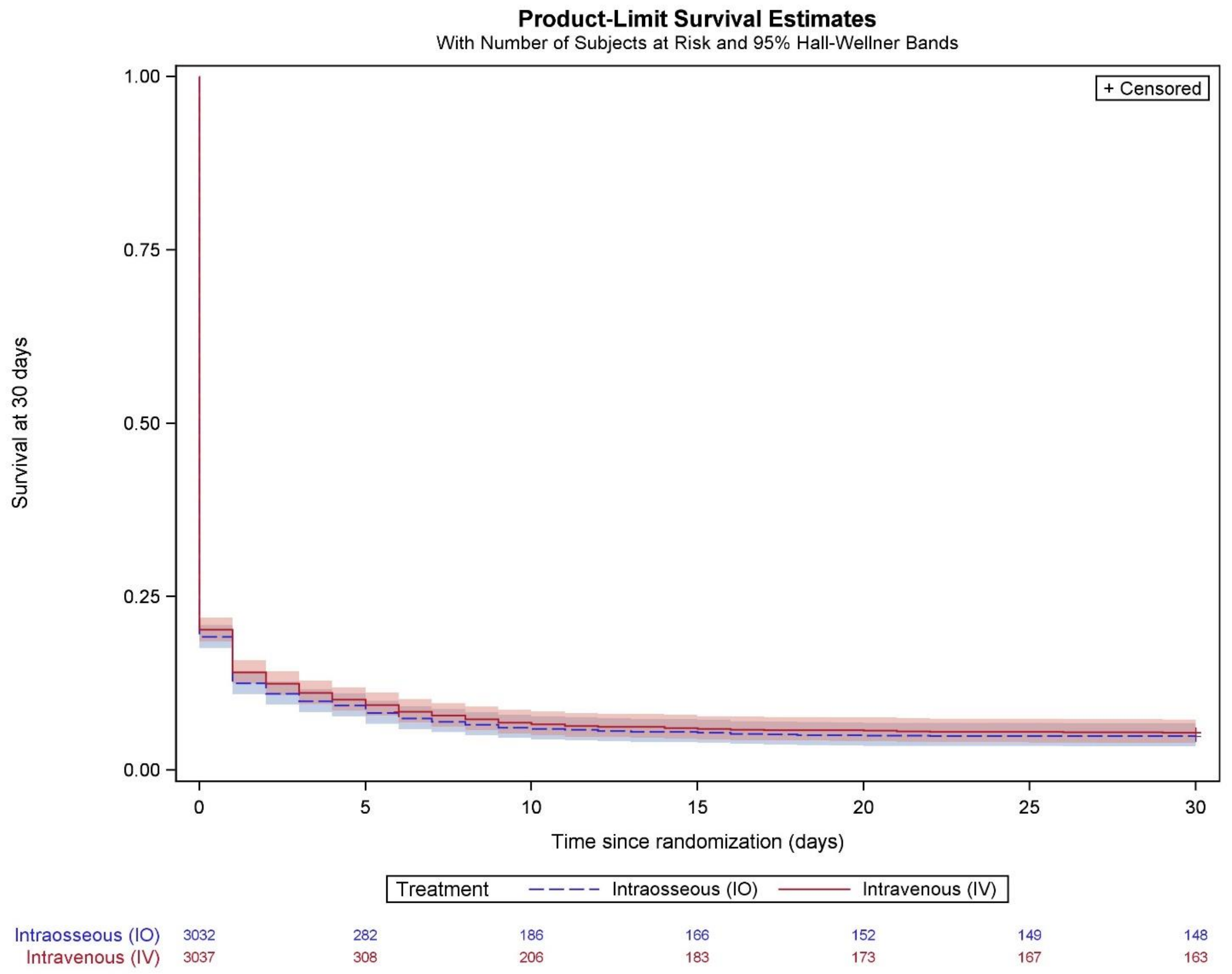

Figure S2: Cumulative incidence curve for time to first ROSC by treatment arm

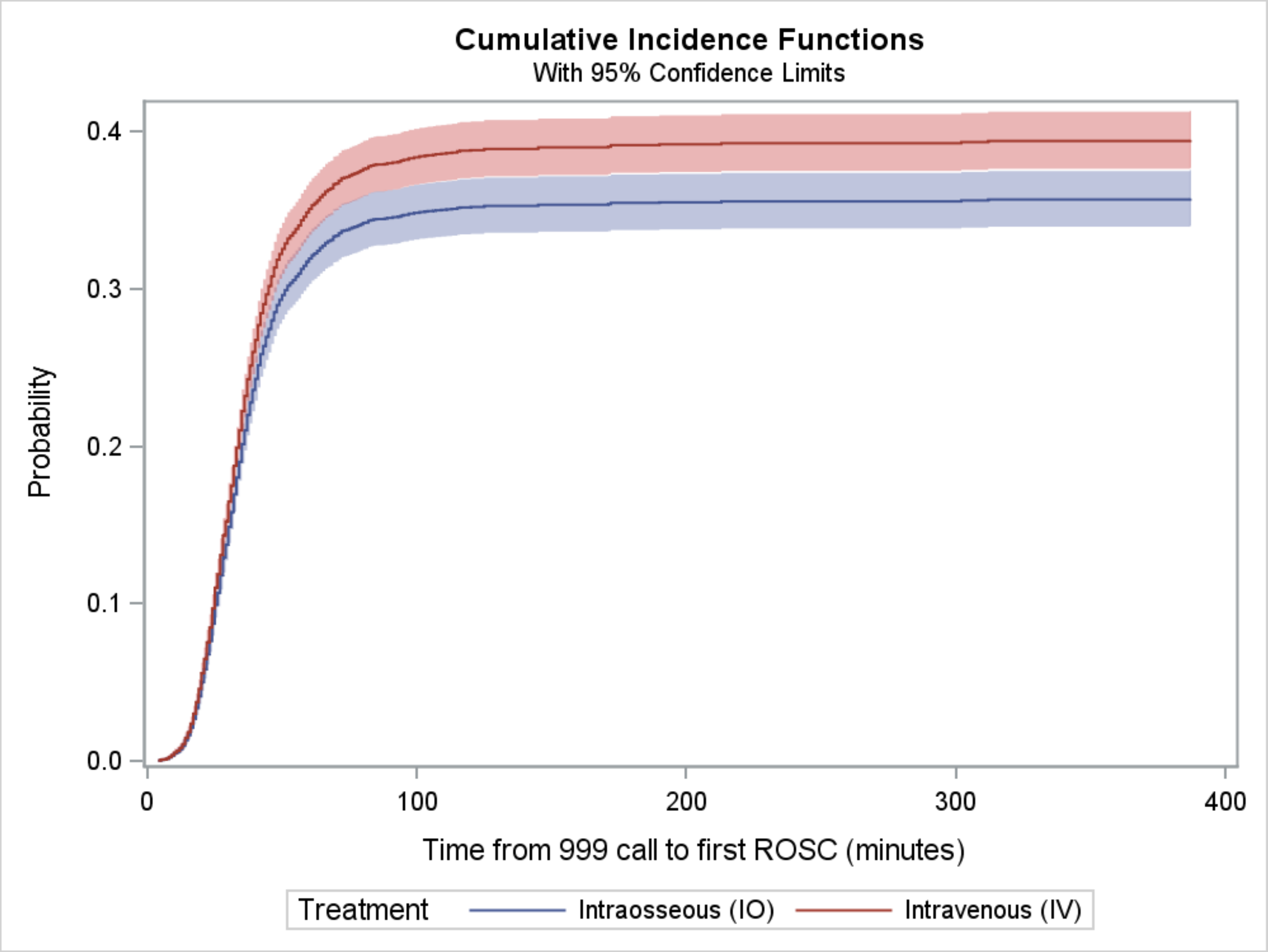

Figure S3: Cumulative incidence curve for time to sustained ROSC at hospital handover by treatment arm

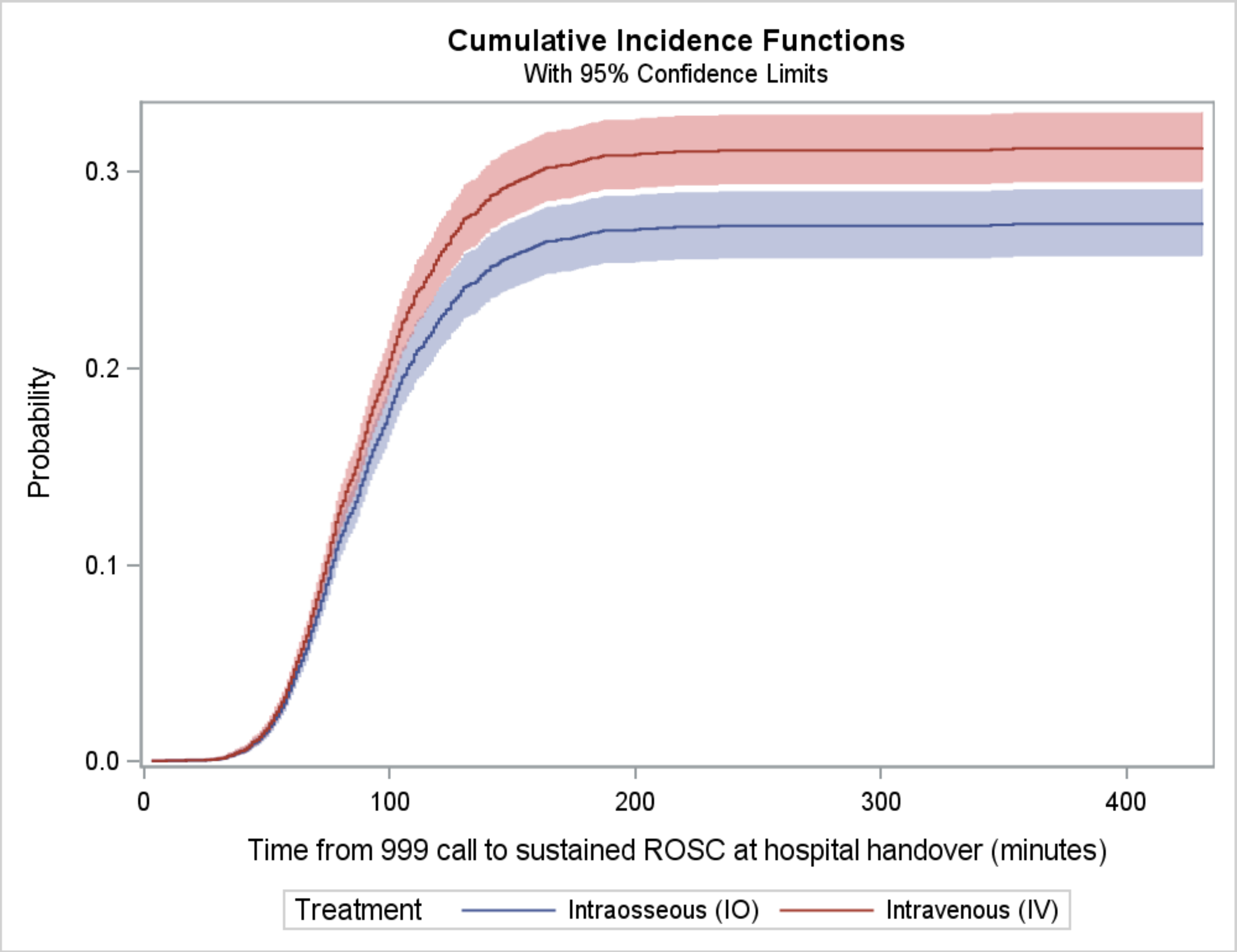

Figure S4: Kaplan Meier curve for survival to hospital discharge by treatment arm

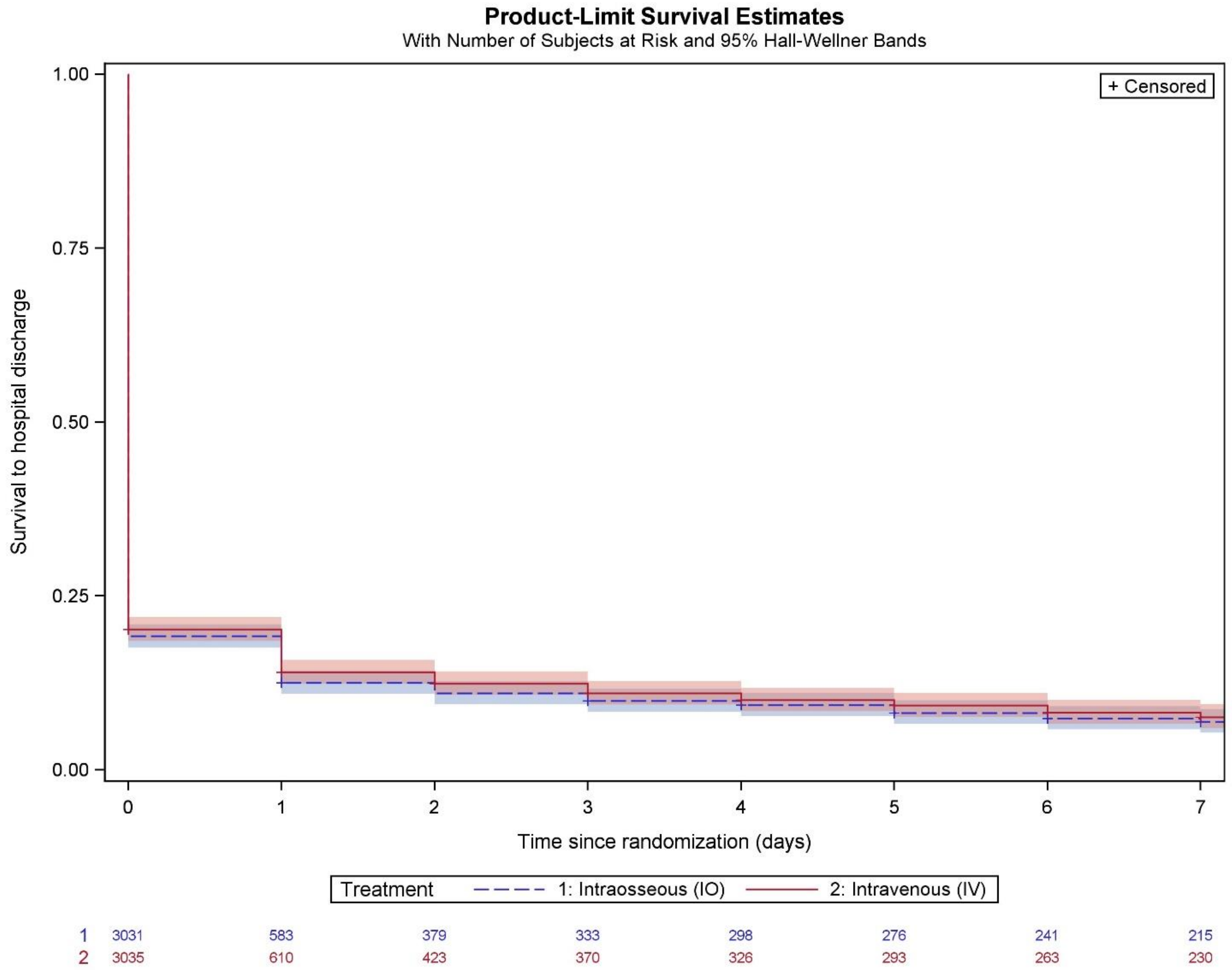

Figure S5: Summary of unadjusted subgroup analyses for primary outcome

Subgroup	IO	IV	Absolute difference (95% CI)	OR (95% CI)
Age				
Mean age (68.1 years old)				0.88 (0.68, 1.15)
Sex				
Male	111/1933 (5.7%)	106/1945 (5.4%)	0.3% (-1.2%, 1.7%)	1.06 (0.80, 1.39)
Female	26/1061 (2.5%)	45/1047 (4.3%)	-1.8% (-3.4%, -0.3%)	0.56 (0.34, 0.91)
Witnessed by				
Not witnessed	23/1164 (2.0%)	26/1108 (2.3%)	-0.4% (-1.6%, 0.8%)	0.84 (0.48, 1.48)
By EMS	15/193 (7.8%)	19/183 (10.4%)	-2.6% (-8.4%, 3.2%)	0.73 (0.36, 1.48)
By bystander	96/1637 (5.9%)	109/1697 (6.4%)	-0.6% (-2.2%, 1.1%)	0.91 (0.68, 1.20)
Bystander CPR				
No	35/892 (3.9%)	42/850 (4.9%)	-1.0% (-3.0%, 0.9%)	0.79 (0.50, 1.24)
Yes	98/2082 (4.7%)	112/2139 (5.2%)	-0.5% (-1.8%, 0.8%)	0.89 (0.68, 1.18)
Initial rhythm				
Shockable	103/558 (18.5%)	117/629 (18.6%)	-0.1% (-4.6%, 4.3%)	0.99 (0.74, 1.33)
Non-shockable	30/2411 (1.2%)	37/2356 (1.6%)	-0.3% (-1.0%, 0.3%)	0.79 (0.49, 1.28)
Initial etiology				
Medical	115/2475 (4.6%)	134/2474 (5.4%)	-0.8% (-2.0%, 0.4%)	0.85 (0.66, 1.10)
Non-medical	10/200 (5.0%)	9/199 (4.5%)	0.5% (-3.7%, 4.7%)	1.11 (0.44, 2.80)
Time from call to EMS arrival				
Median time (8.0 minutes)				0.94 (0.83, 1.06)

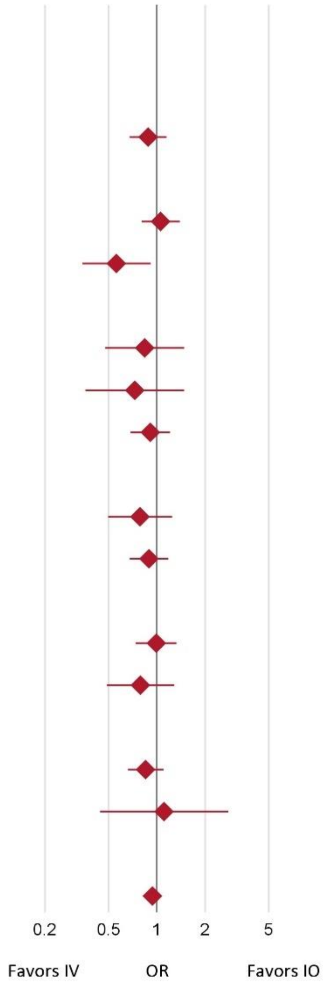

Note: EMS denotes emergency medical service; CPR denotes cardiopulmonary resuscitation. Confidence interval widths have not been adjusted for multiplicity and may not be used in place of hypothesis testing.

Figure S6: Survival with a Favourable Neurological Outcome at Hospital Discharge (Modified Rankin Score)

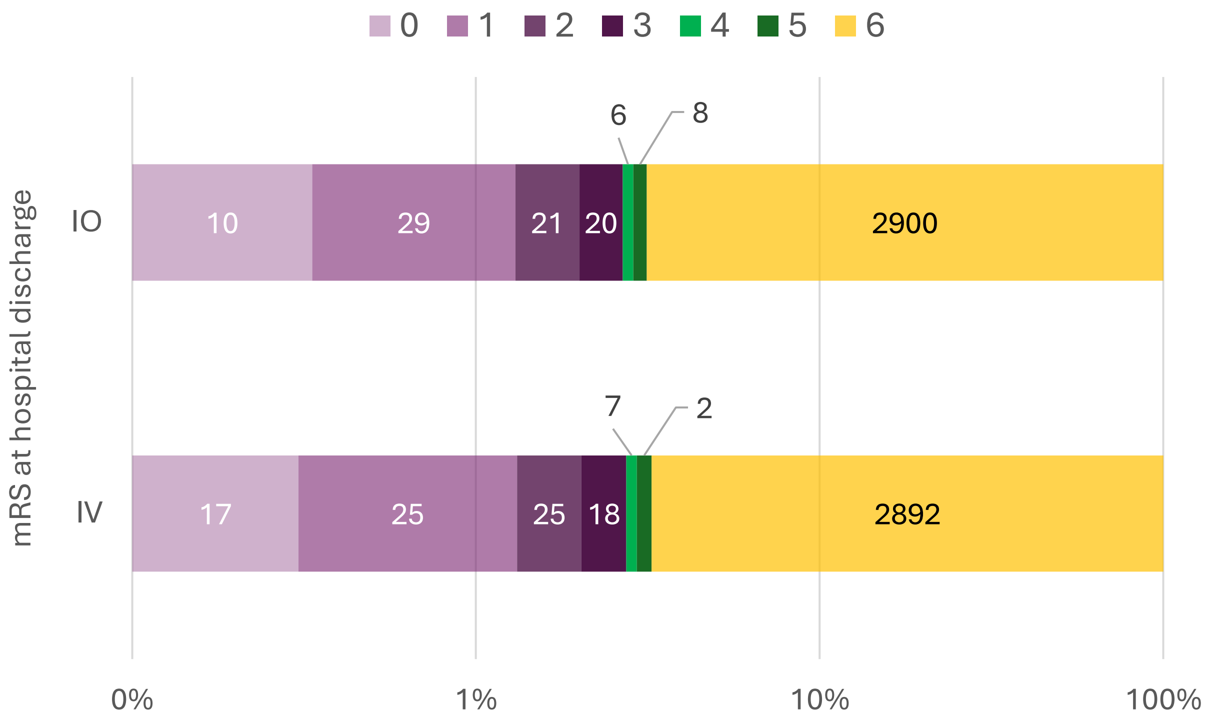

Note: Figure shows the distribution of patients by modified Rankin Score at hospital discharge. Modified Rankin Score is assessed on a 7-point scale from 0 to 6, namely: 0- No symptoms, 1- No significant disability, 2- Slight disability, 3- Moderate disability, 4- Moderate severe disability, 5- Severe disability, 6- Dead. A score of 0-3 is categorized as a favorable neurological outcome.[15] A modified Rankin Score of 3 or less is categorized as a favorable neurological outcome. The data are presented on a $\log_{10}$ scale of the percentages of patients in each group. IO denotes intraosseous; IV denotes intravenous

Figure S7: Tipping point analysis of survival at 30 days

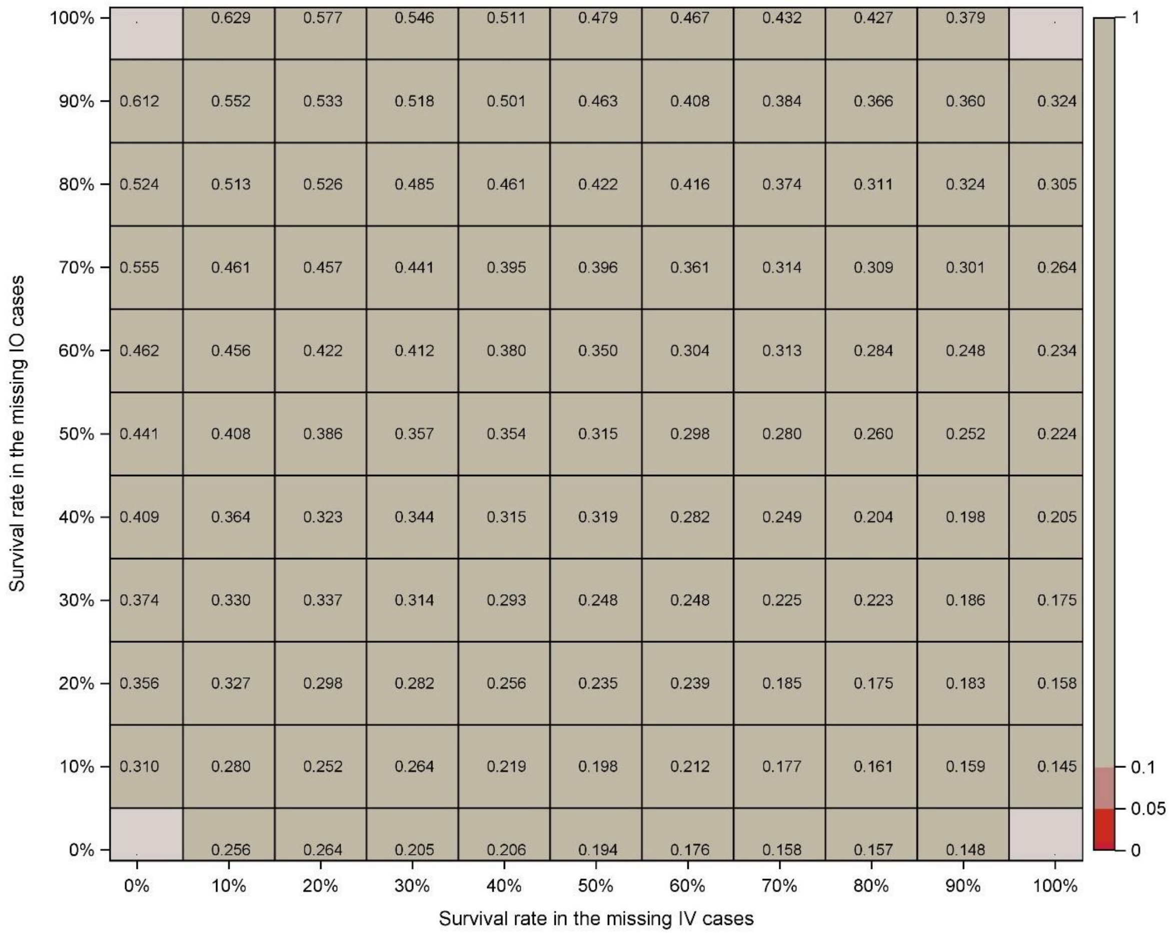

Note: Missing data are assumed to have 0% to 100% survival rate. 10 imputed datasets were generated by Bernoulli sampling approach for each pair of rates to estimate to test the significance of the conclusion.[16] Estimated p values are shown for each pair of rates. As the unadjusted analysis showed a non-significant result, a significant result can thus reverse the conclusion. Any significant results in the tipping point analysis are highlighted in red. Estimation for the extreme scenarios (blank cells where 0% or 100% in either arm) was not made due to the invariance of the rates of the imputed data in one or both arms.

Figure S8: Rate of survival at 30 days over the recruitment period by treatment arm

-

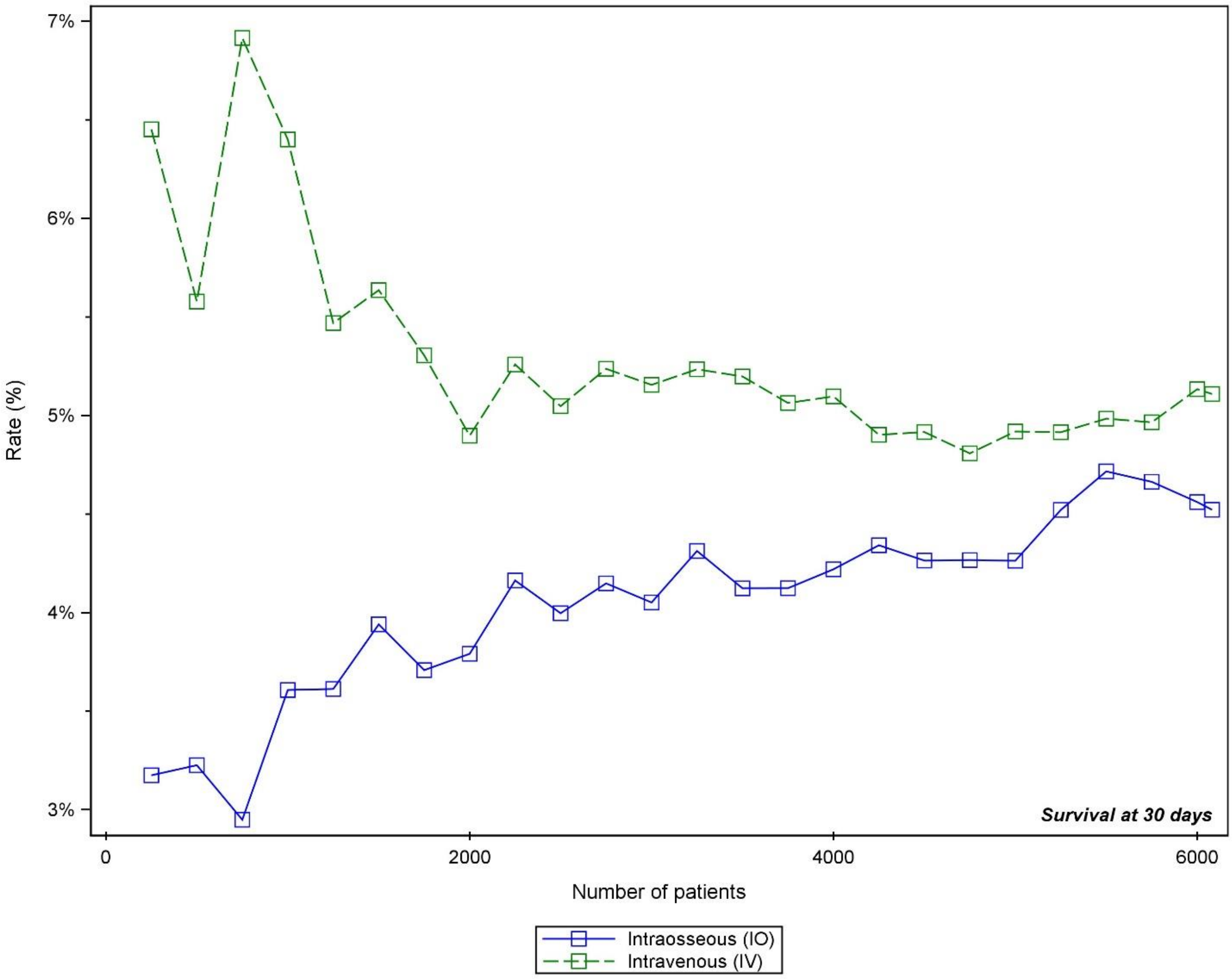

Note: all rates were summarized cumulatively for every 250 recruited patients (treatment combined).

Figure S9: Rate of return of spontaneous circulation at any time over the recruitment period by treatment arm

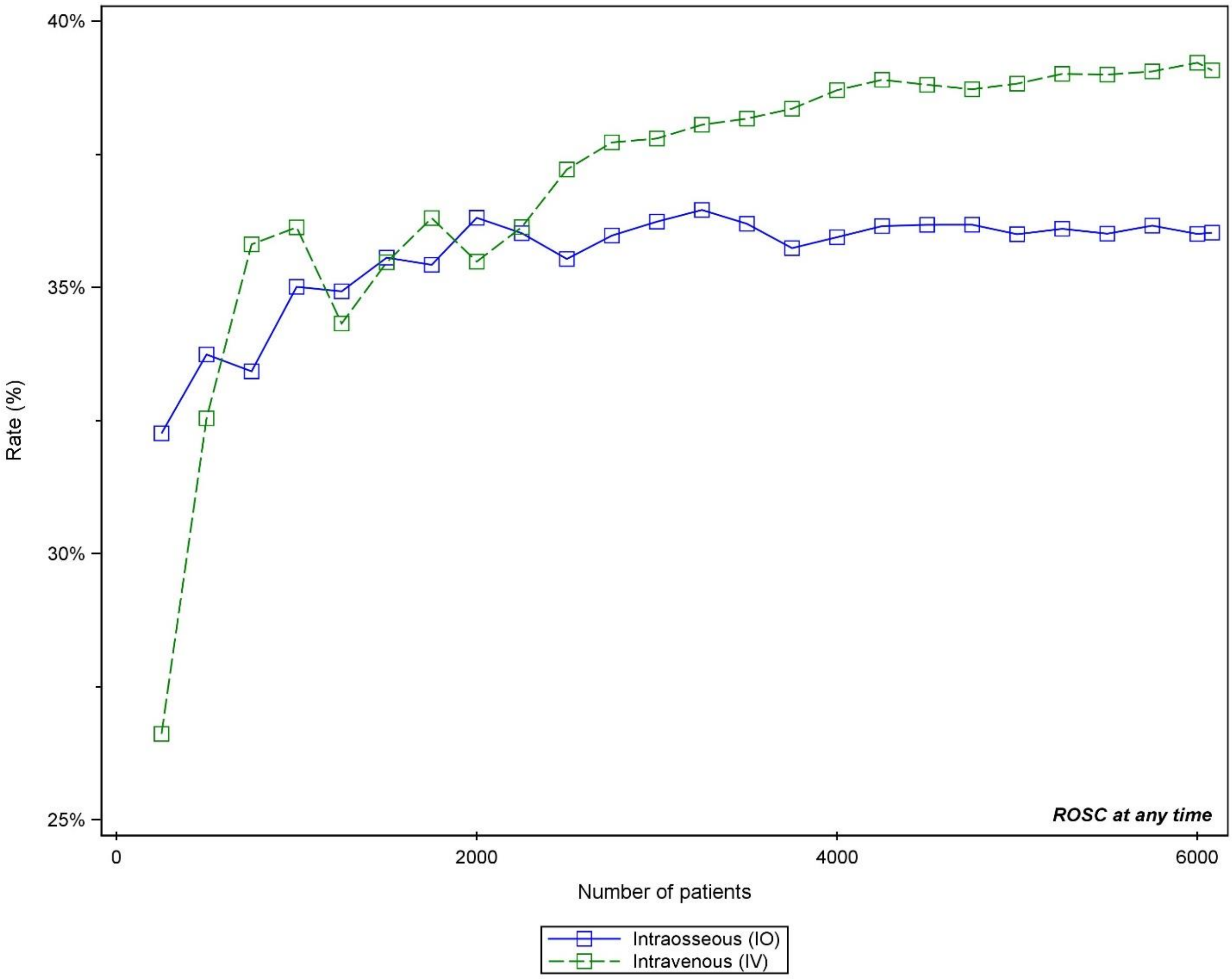

Note: all rates were summarized cumulatively for every 250 recruited patients (treatment combined).

Figure S10: Rate of first attempt success at vascular access over the recruitment period by treatment arm

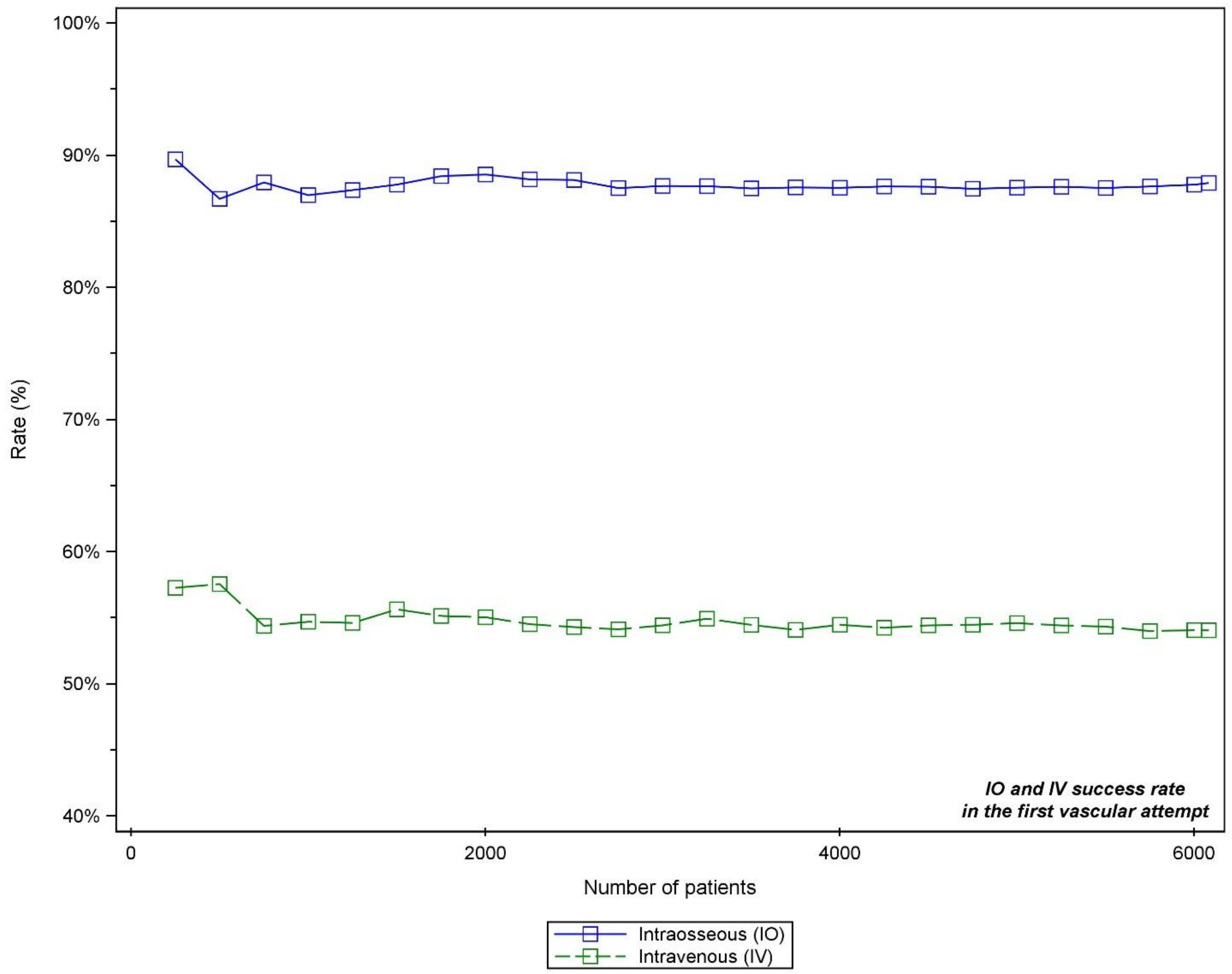

Note: all rates were summarized cumulatively for every 250 recruited patients (treatment combined). For each route, the lines plot the percentage of patients that received successful vascular access via that route at the first attempt.

Supplemental tables

Table S1: Overview of participating centres and recruitment

	Number OHCA treated in 2022	Recruitment start date	Date of last randomisation	Recruitment months	Number randomized*	Average recruits per month
Emergency medical service 1	340	14/02/2023	26/06/2024	16	70	4.4
Emergency medical service 2	3667	14/06/2022	27/06/2024	25	186	7.4
Emergency medical service 3	2723	16/02/2022	30/06/2024	29	694	24.0
Emergency medical service 4	4532	01/03/2022	21/06/2024	28	2339	83.5
Emergency medical service 5	2323	07/03/2022	30/06/2024	28	330	11.8
Emergency medical service 6	4041	14/03/2022	30/06/2024	28	377	13.5
Emergency medical service 7	2666	11/11/2021	30/06/2024	32	736	23.0
Emergency medical service 8	2877	21/07/2022	18/06/2024	24	176	7.3
Emergency medical service 9	3618	09/05/2022	20/06/2024	26	583	22.4
Emergency medical service 10	3596†	08/03/2022	19/06/2024	28	183	6.5
Emergency medical service 11	4025	08/03/2022	19/06/2024	28	422	15.1

Key- OHCA- out-of-hospital cardiac arrest; EMS= Emergency medical services
†- Data is for period April 2022-March 2023.
*Including 14 participants randomized in error.

Table S2: Screening log reasons for non-randomization by site

Site	Total Screened	Total non-enrolment	Known or apparent <18-years	Known or apparent pregnancy	Already had vascular access
ALL EMERGENCY MEDICAL SERVICES	10723	4627	651	62	3914
Emergency medical service 1	376	306	14	0	292
Emergency medical service 2	272	86	0	0	86
Emergency medical service 3	1259	565	9	0	556
Emergency medical service 4	3972	1633	132	57	1444
Emergency medical service 5	366	36	12	0	24
Emergency medical service 6	671	294	46	1	247
Emergency medical service 7	1127	391	122	0	269
Emergency medical service 8	321	145	9	0	136
Emergency medical service 9	1304	721	79	2	640
Emergency medical service 10	303	120	46	0	74
Emergency medical service 11	752	330	182	2	146

Table S3: Participant ethnicity

	Intraosseous (IO) route (N=3040)	Intravenous (IV) route (N=3042)	Total (N=6082)
Ethnicity- no (%)			
White	1376 (45.3%)	1358 (44.6%)	2734 (45.0%)
Mixed	14 (0.5%)	11 (0.4%)	25 (0.4%)
Asian	105 (3.5%)	99 (3.3%)	204 (3.4%)
Black	63 (2.1%)	60 (2.0%)	123 (2.0%)
Other	35 (1.2%)	25 (0.8%)	60 (1.0%)
Not stated or missing	1447 (47.6%)	1489 (48.9%)	2936 (48.3%)

Table S4: Patient status from prior to hospitalization to the end of study

PATIENT STATUS	Intraosseous (IO) route (N=3040)	Intravenous (IV) route (N=3042)	TOTAL
Status at pre-hospital			
Screened	-	-	10723
Met exclusion criteria	-	-	4627
Randomized	3048	3048	6096
Randomized in error	8	6	14
Allocated treatment	3040 (100%)	3042 (100%)	6082 (100%)
Transported to hospital	1024 (33.7%)	1136 (37.3%)	2160 (35.5%)
Achieved ROSC at anytime	1092 (35.9%)	1186 (39.0%)	2278 (37.5%)
Achieved sustained ROSC at hospital handover	654 (21.5%)	744 (24.5%)	1398 (23.0%)
Survived to hospital discharge	112 (3.7%)	120 (3.9%)	232 (3.8%)
Died in hospital	884 (29.1%)	986 (32.4%)	1870 (30.7%)
Still in hospital	28 (0.9%)	30 (1.0%)	58 (1.0%)
- Withdrawal during hospital stay	0 (0.0%)	1 (0.0%)	1 (0.0%)
Status at 30 days			
Survived	137 (4.5%)	155 (5.1%)	292 (4.8%)
Died	2893 (95.2%)	2879 (94.6%)	5772 (94.9%)
Missing	10 (0.3%)	8 (0.3%)	18 (0.3%)
- Withdrawal from the trial before 30 days	0 (0.0%)	1 (0.0%)	1 (0.0%)

Note: patients may stay in ICU/hospital for more than the fixed follow-up timepoints. Hence, the numbers in hospital stay may not add up to match the numbers in the fixed follow-up.

Table S5: Randomization balance by emergency medical service

	Intraosseous (IO) route (N=3040)	Intravenous (IV) route (N=3042)	TOTAL
Emergency medical service 1	34 (48.6%)	36 (51.4%)	70
Emergency medical service 2	86 (46.2%)	100 (53.8%)	186
Emergency medical service 3	350 (50.5%)	343 (49.5%)	693
Emergency medical service 4	1163 (49.7%)	1176 (50.3%)	2339
Emergency medical service 5	176 (53.5%)	153 (46.5%)	329
Emergency medical service 6	186 (49.6%)	189 (50.4%)	375
Emergency medical service 7	377 (51.4%)	356 (48.6%)	733
Emergency medical service 8	92 (52.3%)	84 (47.7%)	176
Emergency medical service 9	284 (49.1%)	295 (50.9%)	579
Emergency medical service 10	85 (46.7%)	97 (53.3%)	182
Emergency medical service 11	207 (49.3%)	213 (50.7%)	420

Table S6: Protocol deviations, violations, and withdrawal by treatment arm

	REASON	Intraosseous (IO) route (N=3040)	Intravenous (IV) route (N=3042)	TOTAL
Deviations	Consent	2 (0.1%)	1 (0.0%)	3 (0.0%)
	Data	26 (0.9%)	19 (0.6%)	45 (0.7%)
	Document Design & Management	0 (0.0%)	1 (0.0%)	1 (0.0%)
	Eligibility & Randomization	7 (0.2%)	1 (0.0%)	8 (0.1%)
	Other	0 (0.0%)	1 (0.0%)	1 (0.0%)
	Staff Delegation & Oversight	1 (0.0%)	1 (0.0%)	2 (0.0%)
	Total	36 (1.2%)	24 (0.8%)	60 (1.0%)
Violations	Consent	1 (0.0%)	0 (0.0%)	1 (0.0%)
	Data	1 (0.0%)	0 (0.0%)	1 (0.0%)
	Eligibility & Randomization	5 (0.2%)	2 (0.1%)	7 (0.1%)
	Total	7 (0.2%)	2 (0.1%)	9 (0.1%)
Withdrawal	Withdrawn from patient reported outcome measures	9 (0.3%)	8 (0.3%)	17 (0.3%)
	Withdrawn from collection and data linkage	6 (0.2%)	7 (0.2%)	13 (0.2%)
	Withdrawal from future approved research	6 (0.2%)	8 (0.3%)	14 (0.2%)
	Withdrawn completely from follow-up	3 (0.1%)	4 (0.1%)	7 (0.1%)

Table S7: Non-compliance by treatment arm

REASON	AMBULANCE SERVICE	Intraosseous (IO) route (N=3040)	Intravenous (IV) route (N=3042)
Non-compliance	Emergency medical service 1	0 (0.0%)	0 (0.0%)
	Emergency medical service 2	3 (0.1%)	1 (0.0%)
	Emergency medical service 3	1 (0.0%)	0 (0.0%)
	Emergency medical service 4	3 (0.1%)	0 (0.0%)
	Emergency medical service 5	2 (0.1%)	0 (0.0%)
	Emergency medical service 6	11 (0.4%)	8 (0.3%)
	Emergency medical service 7	6 (0.2%)	3 (0.1%)
	Emergency medical service 8	2 (0.1%)	1 (0.0%)
	Emergency medical service 9	3 (0.1%)	5 (0.2%)
	Emergency medical service 10	1 (0.0%)	1 (0.0%)
	Emergency medical service 11	11 (0.4%)	7 (0.2%)
	Total	43 (1.4%)	26 (0.9%)
*Discontinuation of treatment**	Emergency medical service 1	2 (0.1%)	0 (0.0%)
	Emergency medical service 2	1 (0.0%)	5 (0.2%)
	Emergency medical service 3	2 (0.1%)	3 (0.1%)
	Emergency medical service 4	53 (1.8%)	48 (1.6%)
	Emergency medical service 5	1 (0.0%)	5 (0.2%)
	Emergency medical service 6	5 (0.2%)	8 (0.3%)
	Emergency medical service 7	10 (0.3%)	20 (0.7%)
	Emergency medical service 8	2 (0.1%)	2 (0.1%)
	Emergency medical service 9	13 (0.4%)	9 (0.3%)
	Emergency medical service 10	0 (0.0%)	0 (0.0%)
	Emergency medical service 11	8 (0.3%)	7 (0.2%)
	Total	97 (3.2%)	107 (3.6%)
*Treatment crossover**	Emergency medical service 1	1 (0.0%)	2 (0.1%)
	Emergency medical service 2	5 (0.2%)	9 (0.3%)
	Emergency medical service 3	28 (0.9%)	58 (1.9%)
	Emergency medical service 4	30 (1.0%)	138 (4.5%)
	Emergency medical service 5	2 (0.1%)	16 (0.5%)
	Emergency medical service 6	9 (0.3%)	30 (1.0%)
	Emergency medical service 7	14 (0.5%)	72 (2.4%)
	Emergency medical service 8	4 (0.1%)	8 (0.3%)
	Emergency medical service 9	24 (0.8%)	44 (1.4%)

	Emergency medical service 10	5 (0.2%)	13 (0.4%)
	Emergency medical service 11	5 (0.2%)	11 (0.4%)
	TOTAL	127 (4.2%)	401 (13.2%)

Note: Non-compliance is defined as any deviation from the protocol (i.e. deviations and violations) and excludes the crossovers. Discontinuation of treatment is defined as no adrenaline is administered. Treatment crossover is defined as the use of the route to which the patient is not randomized before two attempts have been made at the randomized route.

Table S8: Time from emergency medical service arrival to vascular access by rhythm type

	Intraosseous (IO) route (N=3040)	Intravenous (IV) route (N=3042)
Shockable rhythm		
No of patients in analysis	529	597
Time (in minutes)- mean (standard deviation)	11.8 (7.6)	11.7 (7.7)
Time (in minutes)- median (interquartile range)	11 (8, 14)	10 (7, 14)
Non-shockable rhythm		
No of patients in analysis	2291	2223
Time (in minutes)- mean (standard deviation)	13.9 (9.0)	14.8 (10.0)
Time (in minutes)- median (interquartile range)	12 (9, 17)	13 (9, 18)

Table S9: Vascular access success rates

	Intraosseous (IO) route (N=3040)	Intravenous (IV) route (N=3042)	Total (n=6082)
Success at first attempt	2654 (87.3%)	1646 (54.1%)	4300 (70.7%)
Success at second attempt†	190 (6.3%)	298 (9.8%)	488 (8.0%)
Success at second attempt‡	190 (24.5%)	298 (19.4%)	488 (21.1%)

Note †- Includes all randomised patients; ‡-Excludes 3773 patients who had no route attempt information due to success, ROSC, or resuscitation stopped in first attempt. Of the 3773 patients with missing route data at the second attempt, 3662 had successful first attempt and another 81 had no first attempt due to ROSC/resuscitation terminated/Other reasons.

Table S10: Fragility Index of survival at 30 days (Sensitivity analysis)

PRIMARY OUTCOME		Intraosseous (IO) route (OBSERVED)	Intravenous (IV) route (OBSERVED)	Number needed in IO survival to reverse the statistical significance/non-significance	Modified IO	UNADJUSTED ANALYSIS*<br><br>Odds ratio (95% CI)
Survival at 30 days	Yes	137 (4.5%)	155 (5.1%)	54	191 (6.3%)	1.250 (1.005, 1.554)
	No	2893 (95.5%)	2879 (94.9%)		2839 (93.7%)	
	Missing	10	8	18	10	

Note: Confidence interval widths have not been adjusted for multiplicity and may not be used in place of hypothesis testing.

Table S11: Analysis of imputed survival at 30 days and functional outcomes (Sensitivity analysis)

OUTCOME	Imputation method		Intraosseous (IO) route	Intravenous (IV) route	UNADJUSTED ANALYSIS<br><br>OR (95% CI)
Survival at 30 days	MICE		NA	NA	0.887 (0.701, 1.121)
	Best scenario	Yes	147 (4.8%)	163 (5.4%)	0.897 (0.714, 1.128)
		No	2893 (95.2%)	2879 (94.6%)	
	Worst scenario	Yes	137 (4.5%)	155 (5.1%)	0.879 (0.695, 1.112)
		No	2903 (95.5%)	2887 (94.9%)	
Modified Rankin Scale at hospital discharge	MICE		NA	NA	0.952 (0.698, 1.298)
	Best scenario	Favorable	126 (4.1%)	141 (4.6%)	0.890 (0.696, 1.137)
		Unfavorable	2914 (95.9%)	2901 (95.4%)	
	Worst scenario	Favorable	80 (2.6%)	85 (2.8%)	0.940 (0.690, 1.281)
		Unfavorable	2960 (97.4%)	2957 (97.2%)	

Note: OR, odds ratio. MICE: multiple imputation by chained equation. Ten imputations were generated for each outcome. The following variables are included in the imputation model for both outcomes: age, sex, witnessed, bystander CPR, initial rhythm, time from emergency call to drug administration, aetiology of cardiac arrest and ROSC at hospital handover. Imputation was made for 18 (10 in IO) missing survival at 30 days and 102 (46 in IO) missing mRS at hospital discharge. Best scenario: all missing functional (survival) data will be imputed as favorable (alive) outcome. Worst scenario: all missing functional (survival) data will be imputed as unfavorable (deceased) outcome. Confidence interval widths have not been adjusted for multiplicity and may not be used in place of hypothesis testing.

Table S12: Inverse probability of censoring weighted analysis of survival at 30 days (sensitivity analysis)

PRIMARY OUTCOME	UNADJUSTED ANALYSIS OR (95% CI)	ADJUSTED ANALYSIS* OR (95% CI)
Survival at 30 days	0.811 (0.635, 1.050)	0.979 (0.703, 1.361)

Note: IPCW, Inverse probability of censoring weights. Crossovers are considered as censored patients. OR, odds ratio. *- analysis is adjusted for age, sex, witnessed, bystander CPR, initial rhythm, time from emergency call to drug administration, aetiology of cardiac arrest. Confidence interval widths have not been adjusted for multiplicity and may not be used in place of hypothesis testing.

Table S13: Adjusted outcome analyses using alternative time covariates (post-hoc sensitivity analyses)

	Odds ratio/ hazard ratio/ mean difference (95% confidence interval)			
	**Unadjusted analysis**	**Adjusted (primary analysis)***	**Adjusted (post-hoc model one)****	**Adjusted (post-hoc model two)*****
Primary outcome				
**Survival at 30 days**	0.880 (0.695, 1.113)	0.945 (0.676, 1.322)	0.884 (0.671, 1.165)	0.893 (0.679, 1.176)
Secondary outcomes				
**ROSC at any time**	0.878 (0.791, 0.974)	0.863 (0.765, 0.974)	0.880 (0.783, 0.990)	0.883 (0.785, 0.992)
**Time to ROSC**	0.896 (0.823, 0.975)$^{\Delta}$	0.889 (0.808, 0.979)$^{\Delta}$	0.903 (0.824, 0.989)$^{\Delta}$	0.908 (0.829, 0.995)$^{\Delta}$
**Sustained ROSC at hospital handover**	0.848 (0.752, 0.956)	0.853 (0.741, 0.983)	0.866 (0.756, 0.991)	0.872 (0.762, 0.997)
**Survival to hospital discharge**	0.931 (0.716, 1.210)	0.996 (0.679, 1.461)	0.963 (0.709, 1.309)	0.967 (0.713, 1.312)
**Length of hospital stay**				
**Patients who survived**	3.122 (-4.698, 10.942)	7.681 (-4.392, 19.754)	3.572 (-4.744, 11.888)	3.554 (-4.666, 11.773)
**Patients who died**	-0.229 (-0.483, 0.024)	-0.178 (-0.454, 0.098)	-0.233 (-0.521, 0.055)	-0.203 (-0.495, 0.089)
**Favourable Neurological Outcome at Hospital Discharge**	0.937 (0.687, 1.277)	0.914.567, 1.474)	0.947 (0.664, 1.353)	0.949 (0.666, 1.353)

Note:
ROSC- return of spontaneous circulation
All outcomes are presented as odds ratio and 95% confidence interval, except for time to ROSC (presented as hazard ratio and 95% confidence intervals) and length of hospital stay (presented as mean difference and 95% confidence interval).
The unadjusted analysis and adjusted (primary analysis) are as presented in the manuscript and are presented here to aid comparison across models. The two post-hoc models are presented to mitigate any causal association between time to drug administration and outcome as time to drug administration was included as a covariate in the primary adjusted analysis. In post-hoc model one, the covariate time from emergency call to drug administration is replaced by time from emergency call to arrival at scene. In post-hoc-model, the covariate time from emergency call to drug administration has been removed and not replaced.
Modified Rankin Score is assessed on a 7-point scale from 0 to 6, namely: 0- No symptoms, 1- No significant disability, 2- Slight disability, 3- Moderate disability, 4- Moderate severe disability, 5- Severe disability, 6- Dead. A score of 0-3 is categorized as a favorable neurological outcome.[15]
Confidence interval widths have not been adjusted for multiplicity and may not be used in place of hypothesis testing.

*- Model adjusted for age, sex, witness status (EMS versus bystander), bystander CPR (yes/no), initial rhythm (shockable versus non-shockable), time from emergency call to drug administration, etiology of cardiac arrest (medical versus non-medical).
**- Model adjusted for age, sex, witness status (EMS versus bystander), bystander CPR (yes/no), initial rhythm (shockable versus non-shockable), time from emergency call to arrival at scene, etiology of cardiac arrest (medical versus non-medical).
***- Model adjusted for age, sex, witness status (EMS versus bystander), bystander CPR (yes/no), initial rhythm (shockable versus non-shockable), etiology of cardiac arrest (medical versus non-medical).
Δ Cause-specific hazard function was used to estimate the hazards of ROSC. Death before any ROSC is considered as a competing risk. Proportional hazard assumption was not violated for both unadjusted and adjusted analyses.

Table S14: Summary of Adverse Events

ADVERSE EVENTS		Intraosseous (IO) route	Intravenous (IV) route	TOTAL
**Relationship to trial intervention (causality)**	Definitely	0 (0.0%)	0 (0.0%)	0 (0.0%)
	Probably	1 (0.0%)	0 (0.0%)	1 (0.0%)
	Possibly	0 (0.0%)	0 (0.0%)	0 (0.0%)
**Time of event**	At cardiac arrest	1 (0.0%)	0 (0.0%)	1 (0.0%)
	During hospital stay	0 (0.0%)	0 (0.0%)	0 (0.0%)
	At hospital discharge	0 (0.0%)	0 (0.0%)	0 (0.0%)
**N of AE**		1	0	1

Table S15: List of Adverse Events by treatment arm

**Number**	**Details of adverse event**
**Intraosseous (IO) route**	
1	Patient complaining of ongoing dull ache-like pain at proximal left tibia, felt on weight bearing and moderate exercise.
**Intravenous (IV) route**	
None recorded	Not applicable

Table S16: List of serious Adverse Events by treatment arm

**Number**	**Details of adverse event**
**Intraosseous (IO) route**	
None reported	Not applicable
**Intravenous (IV) route**	
None recorded	Not applicable

References

1. Brown SN, Walker A, James C, Wrigley F, Joint Royal Colleges Ambulance Liaison C, Association of Ambulance Chief E. JRCALC clinical guidelines 2022. 2022 edition. Bridgwater, England: Class Professional Publishing; 2022.

2. Hawkes C, Booth S, Ji C, et al. Epidemiology and outcomes from out-of-hospital cardiac arrests in England. Resuscitation 2017;110:133-140.

3. Vadeyar S, Buckle A, Hooper A, et al. Trends in use of intraosseous and intravenous access in out-of-hospital cardiac arrest across English ambulance services: A registry-based, cohort study. Resuscitation 2023;191:109951.

4. Soar J, Böttiger BW, Carli P, et al. European Resuscitation Council Guidelines 2021: Adult advanced life support. Resuscitation 2021;161:115-151.

5. Soar J, Berg KM, Andersen LW, et al. Adult Advanced Life Support: 2020 International Consensus on Cardiopulmonary Resuscitation and Emergency Cardiovascular Care Science with Treatment Recommendations. Resuscitation 2020;156:A80-A119.

6. Davies H, Shakur H, Padkin A, Roberts I, Slowther AM, Perkins GD. Guide to the design and review of emergency research when it is proposed that consent and consultation be waived. Emerg Med J 2014;31(10):794-5.

7. Staniszewska S, Brett J, Simera I, et al. GRIPP2 reporting checklists: tools to improve reporting of patient and public involvement in research. BMJ 2017;358:j3453.

8. Perkins GD, Ji C, Deakin CD, et al. A Randomized Trial of Epinephrine in Out-of-Hospital Cardiac Arrest. New England Journal of Medicine 2018;379(8):711-721.

9. Perkins GD, Lall R, Quinn T, et al. Mechanical versus manual chest compression for out-of-hospital cardiac arrest (PARAMEDIC): a pragmatic, cluster randomised controlled trial. The Lancet 2015;385(9972):947-955.

10. Pocock H, Deakin CD, Lall R, et al. Prehospital optimal shock energy for defibrillation (POSED): A cluster randomised controlled feasibility trial. Resuscitation Plus 2024;17:100569.

11. The Editors. Striving for Diversity in Research Studies. New England Journal of Medicine 2021;385(15):1429-1430.

12. Scobie S, Spencer J, Raleigh V. Research report: June 2021- Ethnicity coding in English health service datasets. London: 2021. (https://www.nhsrho.org/wp-content/uploads/2023/05/Nuffield-Trust-Ethnicity-coding_WEB.pdf).

13. Felton T, Pattison N, Fletcher S, Finney S, Walsh T, Dark P. Co-enrolment to UK Critical Care Studies – A 2019 update. Journal of the Intensive Care Society 2020;23(1):53-57.

14. Krige A, Pattison N, Booth M, et al. Co-Enrolment to Intensive Care Studies – A UK Perspective. Journal of the Intensive Care Society 2013;14(2):103-106.

15. Haywood K, Whitehead L, Nadkarni VM, et al. COSCA (Core Outcome Set for Cardiac Arrest) in Adults: An Advisory Statement From the International Liaison Committee on Resuscitation. Resuscitation 2018;127:147-163.

16. Sui Y, Bu X, Duan Y, Li Y, Wang X. Application of Tipping Point Analysis in Clinical Trials using the Multiple Imputation Procedure in SAS, presented at PharmaSUG conference 2023. (https://www.pharmasug.org/proceedings/2023/SD/PharmaSUG-2023-SD-069.pdf).
